# Economic Aspects of Delivering Primary Care Services: An Evidence Synthesis to Inform Policy and Research Priorities

**DOI:** 10.1111/1468-0009.12536

**Published:** 2021-09-02

**Authors:** LORCAN CLARKE, MICHAEL ANDERSON, ROB ANDERSON, MORTEN BONDE KLAUSEN, REBECCA FORMAN, JENNA KERNS, ADRIAN RABE, SØREN RUD KRISTENSEN, PAVLOS THEODORAKIS, JOSE VALDERAS, HANS KLUGE, ELIAS MOSSIALOS

**Affiliations:** ^1^ London School of Economics and Political Science; ^2^ Trinity College Dublin; ^3^ University of Exeter; ^4^ Danish National Centre for Social Research (VIVE); ^5^ Imperial College London; ^6^ World Health Organization Regional Office for Europe (WHO/Europe)

**Keywords:** Economics, Primary Care Services, Coverage, Financing, Service Delivery, Governance, Umbrella Review

## Abstract

**Policy Points**

- The 2018 Declaration of Astana reemphasized the importance of primary health care and its role in achieving universal health coverage. While there is a large amount of literature on the economic aspects of delivering primary care services, there is a need for more comprehensive overviews of this evidence. In this article, we offer such an overview.
- Evidence suggests that there are several strategies involving coverage, financing, service delivery, and governance arrangements which can, if implemented, have positive economic impacts on the delivery of primary care services. These include arrangements such as worker task‐shifting and telemedicine.
- The implementation of any such arrangements, based on positive economic evidence, should carefully account for potential impacts on overall health care access and quality. There are many opportunities for further research, with notable gaps in evidence on the impacts of increasing primary care funding or the overall supply of primary care services.

**Context:**

The 2018 Declaration of Astana reemphasized the importance of primary health care and its role in achieving universal health coverage. To strengthen primary health care, policymakers need guidance on how to allocate resources in a manner that maximizes its economic benefits.

**Methods:**

We collated and synthesized published systematic reviews of evidence on the economic aspects of different models of delivering primary care services. Building on previous efforts, we adapted existing taxonomies of primary care components to classify our results according to four categories: coverage, financing, service delivery, and governance.

**Findings:**

We identified and classified 109 reviews that met our inclusion criteria according to our taxonomy of primary care components: coverage, financing, service delivery, and governance arrangements. A significant body of evidence suggests that several specific primary care arrangements, such as health workers' task shifting and telemedicine, can have positive economic impacts (such as lower overall health care costs). Notably absent were reviews on the impact of increasing primary care funding or the overall supply of primary care services.

**Conclusions:**

There is a great opportunity for further research to systematically examine the broader economic impacts of investing in primary care services. Despite progress over the last decade, significant evidence gaps on the economic implications of different models of primary care services remain, which could help inform the basis of future research efforts.

Strengthening primary health care is key to progress toward universal health coverage (UHC)^1^ and the achievement of the United Nations' Sustainable Development Goals.^2^, ^3^ In 1978, in the Declaration of Alma‐Ata, the World Health Organization (WHO) and the United Nations Children's Fund (UNICEF) coordinated countries' support for improving health for all through primary health care.^4^ In 2018, the Declaration of Astana marked the renewal of this support, emphasizing that comprehensive primary health care should meet people's health needs, systematically address the broader determinants of health, and empower individuals to optimize their health.^5^ The Declaration also emphasized primary care as a central element of integrated health services.^6^

This review focuses on the economic aspects of delivering primary care services, which are a core component of primary health care. From an economic standpoint, primary care services can both prevent the need for more resource‐intensive secondary care services and foster health improvements that can increase people's productivity and economic outputs.^7^ This view and wider economic benefits have been highlighted by several reports and commissions led by international organizations, though such work has often placed the benefits of primary care service investments in the broader context of improving health and strengthening health systems.^8^, ^9^, ^10^, ^11^, ^12^ These standpoints build on seminal work and commentary focused on identifying and acting on the critical role of primary health care in contributing to overall health systems and population health outcomes.^7^, ^13^, ^14^

Despite clear calls to direct more funding to primary care services based on their economic benefit to health systems and broader society,^15^, ^16^, ^17^ the policymakers responding to these calls have faced a complex and large web of evidence to guide the use of additional funds. Oversight of more efficient and more effective ways to use those funds is not easily identifiable or obtainable. This work seeks to offer an overview that extends beyond the previous efforts to review the evidence regarding the economics of delivering primary care services—which focused on specific geographic areas or study methods, thereby limiting their potential comprehensiveness and generalizability.^18^, ^19^

The challenges for policymakers to translate research into policy are exacerbated by the varying extent to which the evidence on different approaches to financing, governing, and delivering primary care services follows a coherent taxonomy.^20^ We applied the rigorous approach offered by an umbrella review of systematic reviews and used a clear taxonomy for sorting the focus and findings of the included reviews. In this way, we have addressed the limitations of previous research and filled the gap of comprehensive overviews in this topic area.^15^, ^21^

## Objectives

Our primary objective was to synthesize the evidence on the economic aspects of delivering primary care services to support policies for funding and planning primary care services. We did this by defining, developing, and drawing on a taxonomy of the main economic arrangements for delivering primary care services.

## Methods

We conducted an umbrella review of existing systematic reviews. Our review had five steps. First, we defined “the economic impacts of delivering primary care services,” and in order to reflect the breadth of our focus and evidence that we identified, we have structured our evidence synthesis around the theme of “economic aspects.” Second, we developed a taxonomy that complemented our proposed definition and informed our search and synthesis. Third, we used umbrella review methods to identify and assess published systematic reviews and meta‐analyses.^22^ Fourth, we categorized and described the available evidence using our taxonomy. Fifth, we synthesized our conclusions to support policymaking and to suggest avenues for further research.

### The Economic Impacts of Delivering Primary Care Services: Definition and Taxonomy

To define what we meant by the “the economic impacts of delivering primary care services,” we incorporated terms used by international agencies, highly cited research on the core dimensions of primary care systems, and a recent scoping review of evidence supporting the economic benefits of primary care services.^4^, ^6^, ^7^, ^18^, ^20^, ^23^, ^24^, ^25^

Box 1 describes our review's definition of the economic impacts of delivering primary care services. Our understanding of these services arose from previous authoritative definitions from academic research and norm‐setting institutions.^5^, ^6^, ^7^ We defined “delivery” by adopting WHO's nomenclature: “service provision or delivery is an immediate output of the inputs into the health system, such as the health workforce, procurement and supplies, and financing.”^26^ Here “delivery” refers to the monetary and nonmonetary value, composition, and arrangement of these “inputs.” We established that “economic impacts” were quantifiable measures related to the current and/or future resources of the users and/or providers of primary care services. However, our focus on economic impacts and subsequent findings should be considered in the context of potential trade‐offs with other important facets of delivering primary care services. Indeed, individuals' access to, and the quality of, those services may themselves affect long‐term health care costs. Narrowing our focus thus was necessary to ensure that we could feasibly conduct and summarize the findings of a review of such a large body of evidence.

**Box 1. Our Definition**

We developed the following definition of “the economic impacts of delivering primary care services,” based on consensus from coauthors and relevant authoritative sources, to guide our search and synthesis of evidence:

Quantifiable outcomes on the current or future resources available to users and providers of health services and that are attributable to the arrangements and resources directly supporting the implementation of primary care services and their functioning.

Primary care services are multidisciplinary health care services that support the primary health care system's core functions of first‐contact care, continuity, coordination, comprehensiveness (including promotive, protective, preventive, curative, rehabilitative, and palliative care), and patient centeredness.

We outline the rationale for this definition and our approach to grouping evidence in the Methods section.

We developed and iteratively refined a taxonomy of the components and subcomponents of the delivery of primary care services (see Table 1). This process involved synthesizing the existing frameworks used by international agencies such as the Operational Framework linked to the Declaration of Astana, examining cross‐country international comparisons of primary care services, and conducting internal peer reviews.^20^, ^26^, ^27^, ^28^, ^29^, ^30^, ^31^, ^32^, ^33^ In line with previous literature and the Declaration of Astana (2018), we defined these services as those delivering the “first contact with health services.” Examples are general practitioners' services, emergency department care, dentistry services, and community pharmacies.^5^, ^34^

**Table 1:** Taxonomy of the Components of the Economic Impacts of Primary Care Service Delivery

**Coverage**	
Arrangements to address: **Costs**: How primary care services are charged for/funded. **Population**: How primary care services are arranged and supported to ensure the target population can get the support they need. **Services**: How primary care services are arranged and supported to ensure a breadth of services are available.	
**Financing**	
Arrangements to improve: **Contracting**: How purchasers and payers contract providers to supply primary care services. **Procurement**: How purchasers and payers procure additional primary care services to supplement government‐run services. **Resource Allocation**: How purchasers and payers allocate finite resources for primary care services and facilities.	
**Service Delivery**	
Workforce arrangements focused on: **Collaboration, Contribution, and Substitution**: The mix of skills and roles of the primary care workforce. **Demand, Supply, and Training**: How the primary care workforce is developed and maintained in different geographic areas. **Support**: How the well‐being and effectiveness of the primary care workforce is supported. Infrastructure arrangements focused on: **Access**: The physical infrastructure in place to support access to primary care services. **Fixed capital assets**: How primary care facilities are planned, built, and maintained. **Resilience measures**: The measures ensuring that primary care services remain responsive during and following a disruptive incident, such as an infectious disease outbreak or extreme weather event. Information technology arrangements focused on: **Communications and remote health technologies**: How communications and remote health technologies support the delivery of primary care services. **Health records and decision supports**: How health information systems integrate the delivery of services and help integrate primary care within the wider health system. **Surveillance and diagnostic tools**: Primary care surveillance measures that monitor the prevalence and burden of disease.	
Patient support arrangements focused on: **Care pathways and service coordination**: How patient pathways are managed throughout the delivery of primary care services and how the integration or coordination of health care providers affects the delivery of health care.	
**Governance**	
Arrangements to improve: **Accountability and evaluation**: How primary care providers are monitored, regulated, and supported to comply with standards and guidance. **Stakeholder engagement**: How patients, providers, and payers are actively engaged in the delivery of primary care services.	

This review's taxonomy focused on the potential economic impacts of different arrangements for, or models of, the delivery of primary care services. This meant that we did not focus on the impacts of specific interventions implemented in primary care, such as the introduction of novel health technologies (specific pharmaceutical drugs or medical devices). We hope this approach will collate information in a way that can help to inform strategic policymaking decisions on how and where to invest in the delivery of primary care services.

### Umbrella Review

We conducted an umbrella review to identify and synthesize quality evidence reviews into one accessible and usable document. Our review methods adhere to those set out in our PROSPERO‐published protocol (CRD42019125040).^35^ This methodology draws on recommendations for conducting umbrella reviews and appraising economic evidence.^36^, ^37^, ^38^ From now on, in this article, we will refer to those publications that met our inclusion criteria as “included reviews,” to publications that did not meet our inclusion criteria as “candidate reviews,” and to studies synthesized by included reviews as “primary research” or “included studies.” We also refer to “reviews of reviews,” that are publications, like this one, that compiled and synthesized published evidence reviews. Supplementary File 1 further describes the processes of identifying and synthesizing reviews.

## Search Strategy

We searched four electronic databases for evidence reviews: the Cochrane Effective Practice and Organization of Care (EPOC) register of systematic reviews (Cochrane Library), EconLit, EMBASE (Ovid), and Medline (Ovid). We searched for English‐language publications published between January 1, 1978 (the year the Declaration of Alma‐Ata was signed) and March 4, 2019.^4^ We also hand‐searched reference lists, including those of publications that were focused on relevant topics but did not meet our eligibility criteria for study methods, such as scoping reviews and overviews of reviews. If publications were inaccessible using available resources, we contacted the authors to request manuscripts and associated materials.

We collated relevant search terms from Cochrane EPOC reviews focused on primary care service delivery, from an evidence gap map of performance measurement and management in primary care, and from internal peer review.^39^, ^40^, ^41^, ^42^, ^43^, ^44^, ^45^, ^46^, ^47^, ^48^, ^49^, ^50^ We then searched electronic databases using terms related to primary care services, economic evidence, and evidence reviews. See Supplementary File 1 for a full list of our search terms.

### Eligibility Criteria

We based our inclusion decisions on a review's reported methods and its inclusion criteria for primary research. For methods, we required that an included review clearly reported an identification as a systematic review or meta‐analysis, an outline of the search strategy, and a structured evidence synthesis.^37^ For interventions and outcomes, an included review had to contain the inclusion criteria used in our taxonomy and definition of “the economic impacts of delivering primary care services,” and it had to be written in English. For populations studied, an included review could not have placed restrictions on primary research that were based on the characteristics of participants. We did not place any restrictions on included reviews' own criteria for primary research designs, use of comparator groups, or geographic focus. If a review generally synthesized evidence regarding an arrangement applied to multiple health care settings, such as primary, secondary, and tertiary care, we included it only when more than half the included studies were conducted in a primary care setting that met our definition.

### Screening and Selection of Studies

Two of us independently screened the titles and abstracts obtained from database searches to identify and review the inclusion candidates. The same two of us then conducted full‐text assessments of those candidates. Any disagreements on study inclusion were resolved by discussion and, if required, by a third of us. One of us, who also conducted screening and full‐text assessments, hand‐searched reference lists to identify additional reviews for screening.

### Quality Assessment

We conducted a quality assessment of a review if it met all other inclusion criteria. Our classifications followed guidance from the SUPPORT Tools for evidence‐informed health policymaking,^51^, ^52^ as the tools offer a comprehensive assessment of quality in a manner that is feasible for an umbrella review that includes more than 100 reviews. Two of us conducted the assessments independently and classified each review as having minor, important, or major limitations. These classifications were based on composite decisions across two domains: the review methods used for identifying studies and the review methods used for analyzing the study findings. See Supplementary File 1 for further details.

### Data Extraction and Synthesis

We designed a standardized form for data extraction, drawing on previously published reviews of reviews of arrangements for delivering health care services (see Supplementary File 1).^36^, ^37^, ^53^, ^54^, ^55^ We piloted the form, refined it through internal peer review, and then applied it to our included reviews. One of us extracted information from all included reviews, and at the same time, another one of us extracted information from a random sample of 10% of included reviews. We used these duplicates to check for accuracy in the abstraction process and to ensure that a standard approach was used. Because we found only minimal discrepancies in the reviewers' extraction forms, we decided that this 10% sample was sufficient for the purposes of this review. Supplementary File 2 lists the screening decisions for full‐text assessments.

We produced a narrative synthesis of those reviews classified as having minor or important limitations. We conducted the synthesis by sorting the reviews, using our taxonomy (Table 1), based on the components and subcomponents of delivering primary care services, which each review focused on. We based this approach on an adaptation of that taken by a similar review of reviews, which examined the delivery arrangements for health systems in low‐income countries.^56^

In some cases, a review's focus covered more than one topic in our taxonomy (e.g., one might cover both financing and workforce arrangements). In such cases, we used the review's primary topic when summarizing the breadth of focus of included reviews (see Supplementary File 3). However, we have placed the findings from other topics covered by such reviews in the relevant section of our narrative synthesis, summary of findings (see Table 2), and summary of gaps in primary research and systematic reviews. For our summary of findings, we categorized the findings of the included reviews based on whether the authors identified that a specific arrangement for delivering primary health care services had a positive, negligible, or negative economic impact (see Table 2). Supplementary File 3 includes a catalog of the syntheses of the included systematic reviews and a summary table of topics about which the included systematic reviews determined that the available evidence was insufficient to reach any conclusions.

**Table 2:** Summary of Findings

Components	Positive Effects	Negligible/Negative Effects
Coverage	• Policies that restrict the total amount an insurance policy will repay for drug purchases (reimbursement restriction policies), combined with cheaper and effective alternative drugs (e.g., generics), can lower costs.^57^	
Financing	• Blended capitation payments (versus fee‐for‐service models) can improve health care providers' workloads and care utilization.^58^ Further evidence suggests that capitation can drive down costs.^59^ • Prospective payment models (e.g,, fixed budgets for a financial year), can improve dentists' clinical activity.^48^ • Pay‐for‐performance (versus fee‐for‐service payments) can raise activity levels of primary care physicians, as well as reduce future emergency admissions, though the mechanisms are complex.^59^, ^60^	
	• Contracting out health services can be a viable option when a government needs additional support for service delivery and can reduce out‐of‐pocket spending for patients seeking curative care.^61^	
	• Providing financial incentives to patients to use lower cost care and requiring copayments for low‐acuity visits or emergency department visits can reduce costs and inappropriate use of health care.^62^, ^63^, ^64^, ^65^	
Service Delivery: Workforce	• Senior physicians working at triage can lower waiting times in emergency departments.^66^ • Nurses in ambulatory care settings can carry out some activities more quickly and for a lower cost than physicians can (e.g., initiating provision of medications).^67^, ^68^ Nurse triage can also reduce a patient's length of stay in an emergency department.^69^ • Community health workers can be a cost‐effective way to supply primary care services in resource‐constrained settings.^70^ • Rural clinical schooling and placement programs can improve rural primary care employment.^71^	**Negligible** • Increasing the proportion of women working in the primary‐care physicians' workforce appear to have a negligible impact on effect on time spent working. Further research on work‐life balance, care giving, and child‐rearing responsibilities is warranted.^72^ • Colocating clinical pharmacist services in primary care clinics appears to have a negligible impact on economic outcomes.^73^
		**Negative** • Video marketing can have a negative impact on rural primary care recruitment.^74^ • Nurse‐led activities in ambulatory care settings can increase time requirements for some services (e.g., consultations).^75^
	• Substituting physicians in primary care consultations by granting nurses greater authority in their practice can improve the supply of primary care services. Advanced‐practice nurses can have a similar impact.^76^, ^77^ • Pharmacists can offer a lower cost alternative to physicians for patient education and medication review.^78^, ^79^ • Peer advisers providing health‐related lifestyle advice can be a cost‐effective way to improve patients' behaviors.^80^	**Negligible** • The impact of pharmacist‐led, nondispensing services appears to have little impact on hospital attendance, admissions, or effectiveness of care.^78^
	• Recruitment campaigns and financial incentives linked to professionals recruited from rural areas, long service obligations, and flexible career options can improve rural primary care employment.^74^ • The use of biomathematical models to predict fatigue or sleepiness in emergency medical service personnel can support better fatigue mitigation protocols and lead to favorable system‐level impacts on costs.^81^ • Tailored strategies for improving professional practice (e.g., addressing competence and standards of practice) can improve care processes.^82^	
Service Delivery: Infrastructure	• Laboratory testing in primary care settings can lower primary care workloads through easier access to test results and shorter turnaround times.^83^	
Service Delivery: Information Technology	• Health and telemedicine can be cost‐effective substitutes for some in‐person primary care services, but interventions should be considered on a case‐by‐case basis.^84^, ^85^	
	• Telephone consultations for triage can reduce the use of in‐person primary care and offer a more cost‐effective alternative to in‐person assessments.^86^ However, further evaluation was recommended to measure effects on repeat visits and resource use. • Real‐time video communication and teledentistry can be cost‐effective substitutes for home care services and conventional dentistry services.^87^ • Computerized decision‐support systems can reduce workloads and costs of care.^88^, ^89^ • Health information exchange, the sharing of digital patient information between health care facilities and systems, can help lower emergency department costs.^90^ • Patients' access to online electronic health records can lower primary‐care physicians' workloads.^91^ • Clinical prediction rules can reduce the inappropriate use of health care use^92^ and quantify patients' characteristics and histories with the aim of simplifying, standardizing, and increasing the accuracy and consistency of clinicians' diagnostic and prognostic assessments and management decisions.	
Service Delivery: Patients' Supports	• Longer primary care consultations appear to be linked to lower use of secondary health care by patients with ambulatory‐sensitive conditions.^93^ Evidence suggests the emphasis should be on how more time can achieved, rather than on setting specific targets for longer consultations. • Advanced‐access scheduling improves the availability of primary care appointments.^94^ • Accountable care organizations like those in the United States can reduce costs and health care use, compared with less integrated approaches to care provision.^95^ • Fee‐for‐service, pharmacist‐led medication reviews can lower hospitalization rates.^96^	**Negligible** • Public health care and private suppliers' initiatives to systematically offer general health checks to the general population appear to have a negligible impact on hospitalization rates.^97^ • Pharmacist‐led medication reconciliation after hospital discharge appears to have a negligible impact on primary care workloads.^98^
	• Care approaches that can save costs and reduce emergency department use include interdisciplinary team–based models of care, timed targets for patient disposition, extended hours of primary care services, case management, individualized care plans and information sharing, and managed care interventions.^58^, ^63^, ^99^, ^100^ • Disease management services, telemedicine, and community health care clinics can reduce unplanned use of health care.^101^ • Certain organizational arrangements (e.g., use of short stay and rapid assessment units)^102^ can reduce the length of stay for and use of secondary care. • Colocation of services in primary care facilities can reduce waiting times and costs.^103^ Specialists' outreach clinics in primary care and rural hospitals can reduce costs to patients and can achieve greater cost‐effectiveness through multifaceted interventions.^46^	**Negligible** • Studies of filter interventions that guide patients through primary care consultation before accessing secondary care, such as primary care gatekeeping, appear to have negligible impacts on emergency department visits.^64^ • Medication synchronization and instalment dispensation appears to have a negligible impact on costs.^105^
	• Educational interventions to improve outpatient referrals from primary to secondary care may improve referral processes and associated resource use.^104^	
Governance	• Time‐based targets combined with redesigned work environments and timescales in emergency departments can improve emergency department processes and reduce waiting times.^106^ • Implementation of clinical guidelines for community pharmacies can lower costs.^107^ • Supporting antimicrobial resistance stewardship in emergency departments can lower medication use.^108^	

The findings presented in gray rows are from reviews that had important limitations (when reviewed for quality assessment), or the authors of the review(s) noted that the certainty of evidence was low or involved significant uncertainty.

**Positive**: Only desirable effects found, such as reduced costs or unnecessary use of health care, and no clear evidence of any undesirable effects, such as worse health outcomes.

**Negligible**: Little or no effect found, with no clear evidence of any desirable or undesirable effects.

**Negative**: Only negative effects found, with little or no evidence of any desirable effects.

### Results

**Figure 1:**
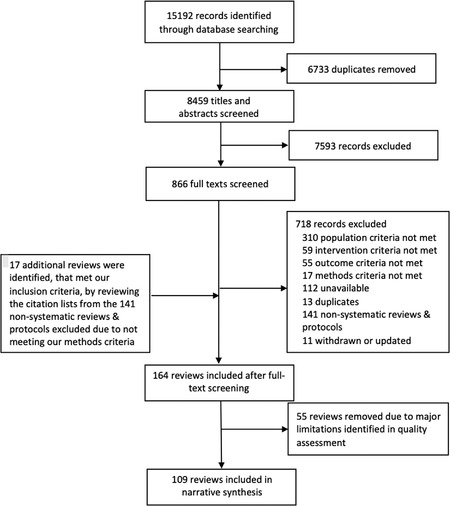
Review Flow Chart for Study Identification and Selection

We screened the titles and abstracts of 8,459 publications from electronic databases. Of these, we found 866 publications that might be relevant. We identified 147 eligible reviews through full‐text screening and another 17 reviews by reviewing the citation lists from the 141 nonsystematic reviews and protocols that did not meet our inclusion criteria, resulting in a total of 164 eligible reviews (see Figure 1). The nonsystematic reviews included scoping reviews. As we explained earlier, more than one of us independently conducted each stage.

We concluded that 67 of these reviews had minor limitations; 42 had important limitations; and 55 had major limitations. Most reviews that we classified as having major limitations were categorized as such because they did not assess the quality of included studies, thereby limiting the extent to which they could evaluate the reliability of those studies' findings. We included the 109 reviews that did not have major limitations in our synthesis.

These 109 reviews covered more than 3,000 studies, although some of them may have appeared in more than one review. Of the reviews that specified the countries from which the study data had come (2,051 studies from 82 reviews), 70% drew on data from the United States (819 studies), the United Kingdom (462 studies), and Australia (147 studies). A small minority of reviews based their conclusions on studies that drew most of their data from countries in Africa, Asia, or Latin America (9 reviews). Eighty‐three of these 109 reviews evaluated primary research published up to 2010, and 39 reviews did so for research published up to 2015.

Using our taxonomy, we split our narrative synthesis into seven areas: coverage, financing, service delivery (workforce, infrastructure, information and technology, and patient pathways), and governance (see Table 1). We did not attempt to identify and assess the quality of individual studies for pragmatic reasons and because the unit of analysis in an umbrella review is the review. Table 2 and subsequent subheadings display key findings from the included reviews sorted by the relevant topic from our taxonomy. We close with a summary of limitations across the included reviews. Supplementary File 3 provides an expanded and individualized summary of all the included reviews, which is complemented by summary tables for the main focus areas of the included reviews, topics about which our findings were uncertain or inconclusive, and research gaps that we identified. These expanded summaries offer further insights into the nuances of the reviews' findings, potential similarities and differences between findings in the included reviews, and more in‐depth descriptions of the topics for which the review findings were uncertain.

#### Coverage

We identified three systematic reviews on arrangements to support coverage of primary care services. Two reviews drew mostly on studies whose data were primarily or entirely collected from countries in North America, and the third review was of studies whose data were primarily or entirely collected from countries in Asia, Africa, the Middle East, and North America.^57^, ^109^, ^110^ The review findings contain some evidence that reimbursement restriction policies such as reference pricing and generic drug policies could be an effective way to lower costs.^57^ For example, one review with important limitations found that eligible studies indicating that reimbursement restriction policies combined with the availability of cheaper and effective alternative drugs could lower costs.^57^ However, another review of the impact of formulary restrictions found that they were associated with less drug use, but the resulting pharmacy cost savings were often offset by increased medical costs.^110^ A review of multiple measures concluded that the economic effects of formulary restrictions in primary care and differential user charges between primary and secondary health care were unclear.^109^

We identified one review of reviews that examined syntheses of financial arrangements across entire health systems in low‐income countries.^111^ It found uncertain evidence regarding the impacts of user fees and community‐based health insurance, and it found other evidence suggesting that cash transfers and vouchers might improve service utilization, but the impacts on costs remained unclear.^111^ Included reviews highlighted that the available evidence suffered from heterogeneous study settings and populations, the use of low‐quality designs (e.g., nonrandomized studies), and poor reporting of implemented arrangements. For example, the conclusions of a review of drug reimbursement arrangements were limited to specific circumstances in which interchangeable drugs were available.^57^

No reviews focusing on changes to the presence or breadth of particular features of primary care services met our inclusion criteria. Also, no reviews which synthesized the evidence on expanding support for primary care services through policies met our inclusion criteria.

### Financing

Eleven systematic reviews focused on arrangements supporting the financing of primary care services.^47^, ^58^, ^59^, ^60^, ^61^, ^112^, ^113^, ^114^, ^115^, ^116^, ^117^, ^118^ Some reviews concluded that there is positive evidence that blended capitation payments and pay‐for‐performance payment mechanisms could reduce overall costs to providers, but their impacts on quality and access of care should be carefully monitored.^58^, ^59^, ^60^ We also found some evidence from reviews focused on supporting better resource allocation in primary care service delivery that certain financial arrangements (i.e., patient financial incentives, user charges for low‐acuity visits, and copayments for emergency department use) reduced costs and health care use.^62^, ^63^, ^64^, ^65^ Review conclusions indicated that the evidence for the total impact of primary care procurement mechanisms was uncertain but that some arrangements for contracting primary care services (i.e., prospective budgets and pay‐for‐performance) could increase certain types of clinical activity. For example, one review found evidence that fixed pharmaceutical budgets were associated with modest reductions in drug use (median relative change was −2.8%).^112^ It is important to note that such financing arrangements should be implemented with a broad lens for potential cost impacts elsewhere in the delivery of primary care and broader health care services, including the overall cost of implementing the new programs.^119^ Although some evidence indicated the potential for reduced out‐of‐pocket spending, the consensus was uncertain about the broader impacts of franchising or contracting out health services.^61^, ^117^, ^118^

The reviews of arrangements for contracting primary care services were almost exclusively made up of studies for which the data were collected from high‐income countries in North America and Europe. We identified three reviews of reviews of the financial arrangements for primary care services in low‐income countries, but the findings were inconclusive and uncertain due to research limitations.^111^, ^120^, ^121^ Generally, the reviews' conclusions were restricted by the heterogeneity of the examined arrangements and limitations in the study methods. For instance, studies of performance‐based financing arrangements often failed to account for selection bias due to voluntary participation and reporting by health care providers or potential confounding when programs were implemented in conjunction with other interventions.^111^, ^117^, ^120^

### Service Delivery

#### Workforce

Thirty‐two systematic reviews were on workforce arrangements for delivering primary care services.^43^, ^66^, ^67^, ^68^, ^70^, ^71^, ^72^, ^73^, ^74^, ^75^, ^76^, ^77^, ^78^, ^79^, ^80^, ^81^, ^82^, ^122^, ^123^, ^124^, ^125^, ^126^, ^127^, ^128^, ^129^, ^130^, ^131^, ^132^, ^133^, ^134^, ^135^, ^136^ The evidence suggested that task shifting among different health care workers may be cost‐effective in certain circumstances, but the overall economic effects often were uncertain, and the available data indicated a possible mix of positive and negative health and economic outcomes. Noted review findings were that hiring community health care workers was a cost‐effective strategy of delivering primary care services in resource‐constrained settings.^70^ We also found some evidence that certain working arrangements for nurses, such as expanding their scope of practice, were associated with increased labor supply, although the overall impacts on cost were often mixed or uncertain.^43^, ^67^, ^68^, ^75^, ^76^, ^77^, ^122^, ^123^, ^124^, ^125^

One review found evidence that placing a senior doctor, rather than a nurse (as usual care), at triage in a hospital emergency department (ED) could reduce lengths of stay and waiting times.^66^ A meta‐analysis of two studies focused on the impact of primary ambulatory care by nurse practitioners found that this arrangement appeared to lower the mean costs per consultation by €6.41 (in 2006 euros).^68^ In comparison, two other similar reviews of this concluded that the evidence for the cost implications of nurses substituting for doctors in primary care was uncertain.^122^, ^137^

Reviews focused on pharmacist‐led nondispensing services had important limitations and mostly uncertain findings. Yet, among them were some optimistic conclusions about potential economic benefits that will require further investigation.^73^, ^78^, ^79^, ^126^, ^127^ Uncertain findings were common among reviews of other primary care workers, such as physician assistants, allied health professionals, and dental auxiliaries,^80^, ^128^, ^129^, ^130^, ^131^ although peer advisers might offer a cost‐effective approach to support if their activities are well targeted to patient behaviors linked to large impacts on health‐related quality of life.^80^ Among the other workforce subtopics covered in the included reviews were some conclusions about specific recruitment and training activities that might have positive economic outcomes.^71^, ^74^, ^133^

Five reviews of reviews examined the impacts of community pharmacy, nurse‐physician collaboration, and nurse retention arrangements. The reviews of reviews on community pharmacies offered a broad scope of nonprescribing interventions and highlighted that while there is supportive evidence that these interventions improve health outcomes, further work is necessary to understand such policies' impacts on resource use and health inequities.^138^, ^139^, ^140^ A review of reviews on nurse‐physician collaboration found that most reviews suggest that nurses provide a clear added value to, and a complementary role in, the delivery of primary care services.^141^ A review of reviews on rural and remote nurse retention interventions found evidence that financial‐incentive programs, peer supports among nurses, information and communication technology support, and rural health careers pathways all could positively influence nurse retention.^142^

The systematic reviews we identified mainly included studies whose data were collected primarily or entirely from countries in North America, Europe, and Australia. Only a small minority of information came from studies whose data were collected primarily or entirely from countries in Asia and Africa. Evidence syntheses from these studies were restricted by the limited scope of costs included and the lack of stable definitions for job roles, such as emergency nurse practitioners.^122^, ^123^, ^124^ Reviews offering recommendations on workforce demand, supply, and training were restricted by small sample sizes, lack of comparison groups, variability in definitions for retention rates, and homogeneous country settings.^74^, ^77^

We identified some positive evidence that measures to support staff working in primary care service delivery, such as tailored interventions to change professional practices involving focus group discussions, interviews, or surveys, could be effective in refining processes of care and improving health outcomes.^143^ These effects were variable, however, and tended to be small to moderate. These reviews were of studies situated mainly in North America and the United Kingdom. Their authors noted that the recommendations were restricted because the reviews drew on small bodies of evidence, had small study sample sizes, and were highly biased.

#### Infrastructure

Two systematic reviews focused on infrastructure of primary care services.^83^, ^144^ One examined the effects of increasing the size of primary care facilities in the United Kingdom and found that the available primary research did not offer any clear conclusions.^144^ Another review focused on surveillance and diagnostic tools found that they could support the workload of physicians conducting laboratory testing in primary care, but the effects on costs remained uncertain.^83^ Evidence regarding health preparedness and planning for primary care services, such as stockpiling supplies to improve resilience,^145^ are often addressed within broader health system interventions and thus fell outside the scope of our review.

The identified reviews primarily included studies whose data were collected from the United Kingdom and the United States and were limited by small sample sizes. We did not find any reviews of reviews focused on infrastructure.

#### Information and Technology

Thirteen of the systematic reviews focused on the use of health information and technology (HIT) in delivering primary care services.^84^, ^85^, ^87^, ^92^, ^146^, ^147^, ^148^, ^149^, ^150^ In general, we found positive evidence that several HIT interventions, such as telemedicine, mHealth, health information exchange, and computerized decision support systems, can lower costs and reduce the use of secondary care.^83^, ^84^, ^85^, ^87^, ^88^, ^89^, ^90^, ^92^ We also found positive evidence that health care decision supports and patient access to electronic health records may reduce inappropriate health care use and improve health workers' workloads, although several of these reviews had important limitations and noted the uncertainty of their findings.^88^, ^89^, ^90^, ^91^, ^92^

Six reviews of reviews examined the impacts of telemedicine and telemonitoring. They had mixed findings and highlighted the need for standardized evaluation and reporting on economic outcomes.^151^, ^152^, ^153^, ^154^, ^155^, ^156^ Reviews offering recommendations about communications and remote health technologies were limited by poor reporting (particularly on costs), large study heterogeneity, and a lack of evidence based on study data from countries in Asia, Africa, Latin America, and the Caribbean. Most of the reviews offering recommendations about health records and decision support systems were based on studies whose data were collected from the United States and the United Kingdom. These studies were limited by small trial sizes, publication biases, randomization biases, and contamination (control group individuals inadvertently being exposed to the intervention).

#### Patient Supports

Forty‐one of the systematic reviews focused on arrangements targeted toward supporting patients, supporting staff activities, and minimizing resource‐intensive activities.^40^, ^42^, ^46^, ^58^, ^62^, ^63^, ^64^, ^65^, ^69^, ^86^, ^93^, ^94^, ^95^, ^96^, ^97^, ^98^, ^99^, ^100^, ^101^, ^102^, ^103^, ^105^, ^157^, ^158^, ^159^, ^160^, ^161^, ^162^, ^163^, ^164^, ^165^, ^166^, ^167^, ^168^, ^169^, ^170^, ^171^, ^172^, ^173^, ^174^, ^175^

We found positive evidence that proactively engaging patients in primary care services through case management, individual care plans, and interdisciplinary team‐based models of care could save costs and reduce emergency department use.^56^, ^58^, ^63^, ^99^, ^100^, ^176^, ^177^, ^178^, ^179^, ^180^ We also found positive evidence that certain organizational arrangements (i.e., short‐stay and rapid‐assessment units) could lessen the use of secondary care and lengthen primary care consultations for patients with ambulatory‐sensitive conditions.^93^, ^102^ We found positive evidence that advanced‐access scheduling in primary care settings, which allows patients to receive same‐day appointment times, was associated with reduced waiting times for appointments, lower no‐show rates, and improvements in continuity.^94^ According to one review, telephone consultations for triage assessments by health professionals appeared to reduce general practitioners' surgery contacts and out‐of‐hour visits.^86^ Integrated care mechanisms like accountable care organizations (ACOs) led to less use of emergency and hospital health care in the United States,^95^ but the impacts of service integration remained unclear in other countries.^168^, ^169^ We found positive evidence that fee‐for‐service, pharmacist‐led medication reviews could significantly lower hospitalization rates (but their impacts on health care costs are uncertain)^96^ and that information leaflets in primary care settings could reduce antibiotic prescription, use, and patients' intention to make a return appointment.^171^ Medication synchronization also can have positive impacts and appears to carry a positive cost‐benefit ratio when associated with the treatment of chronic conditions.^172^

While some patient support mechanisms may have positive economic effects, by means of lower workloads or resource use, several reviews in our study also found evidence that certain patient support mechanisms may have negligible, or even negative, impacts. One review, including five studies on the impact of primary care gatekeeping on emergency department utilization, found only minimal effects.^64^ This review had important limitations and drew findings from studies using data only from the United States, which made it difficult to draw firm conclusions relevant to other health care systems. Another review, including five studies of health care use, concluded that private suppliers' initiatives to systematically offer general health checks to the broader population had little effect on hospitalization rates and might actually lead to unnecessary testing and treatments.^97^ Another review suggested that pharmacist‐led medication reconciliation after hospital discharge was not effective in reducing health care teams' workloads.^98^

The sources of evidence in many of the reviews examining patient support mechanisms provided mixed or uncertain conclusions about the impacts of specific programs and policies. These findings are highlighted further in Supplementary File 3. For example, reviewers could not make conclusions about the effects of altering the length of primary care physician consultations,^42^ whether integrated care in low‐ and middle‐income countries had positive economic impacts,^168^ the economic effects of social prescribing,^170^ the resource outcomes associated with reminder and feedback interventions for medication adherence,^174^ and the cost‐benefit of quality improvement strategies to reduce antibiotic‐prescribing practices.^175^

Ten of the reviews of reviews looked at patient support measures in primary care settings.^56^, ^176^, ^177^, ^178^, ^179^, ^180^, ^181^, ^182^, ^183^, ^184^ The conclusions of our included reviews were based mainly on studies whose data were collected primarily or entirely from countries in North America and Europe, although a minority offered conclusions based on data collected from countries in other regions, such as Asia, Africa, and the Eastern Mediterranean Region. The reviewers pointed out that their recommendations were restricted because the included studies often had small study populations, short follow‐up durations, no cost analysis, and lacked standardized and validated measurements.^126^, ^159^, ^170^ These issues often prevented the reviewers from assessing the feasibility of implementing interventions to address unplanned or frequent usage, despite the observed efficacy for health outcomes.

### Governance

Seven systematic reviews focused on governance arrangements for supporting primary care service delivery.^106^, ^107^, ^108^, ^185^, ^186^, ^187^, ^188^ The included reviews suggested evidence for approaches with positive economic impacts, albeit with caveats, in multiple areas. We found some positive evidence that using targets can reduce waiting times for emergency care services.^106^ We also found positive evidence that clinical guidelines for community pharmacies may lower costs^107^ and that antimicrobial stewardship programs can lower medication use.^108^ Many reviewers pointed out that the impacts of these strategies were contingent on the presence of well‐designed implementation strategies that could account for negative spillover effects, such as the gaming of evaluation metrics. Conclusions from the reviews focused on the effects of managerial supervision arrangements and stakeholder engagement in the delivery of primary care services were less certain.^185^, ^186^, ^187^, ^188^

We identified one review of reviews on governance arrangements for health systems in low‐income countries and two reviews of reviews focused on accountability measures for patients with chronic diseases and mental health issues.^189^, ^190^, ^191^ These reviews included studies that, overall, drew on data from a broad range of countries but usually consisted of small sample sizes, lacked randomization, and had a high risk of bias. Without clear evidence for both health outcomes and professional practice processes, the reviewers could not make robust conclusions.

### Limitations of Included Reviews

Next we describe the four most common limitations of our included evidence.

First, many of the reviews restricted their searches to studies written in English, which may help explain the many studies that used data from the United States, the United Kingdom, and Australia in the included reviews and the corresponding lack of studies that drew on data from other countries.

Second, despite the high‐quality syntheses and reporting of many included systematic reviews, our conclusions were often restricted by the low quality and/or absence of available primary research. The included reviews also consistently noted that many of the reviewed studies did not report health care use and financial costs. Despite requests for the advance publication of economic information (i.e., costs, administrative resource use, potential spillover effects) about health care interventions, reporting gaps in this area persist.^192^ Such evidence gaps were particularly evident in regard to primary care contracting and payment arrangements, which explicitly focus on using monetary arrangements, such as pay‐for‐performance and conditional cash transfer policies.^193^

Third, most of the included reviews stated that the conclusions were restricted because the heterogeneity of the included studies prevented comparisons of characteristics across interventions, measured outcomes, and settings.

Fourth, many reviews were not developed or reported in the context of previous studies or reviews and did not report findings from research on similar topics, thus limiting clarity around how these publications built on the previous literature. In a review of reviews, this omission may also risk “double counting” some of the findings.

## Discussion

### Principal Findings

Our review identified, sorted (using our taxonomy), and synthesized evidence from 109 systematic reviews that can inform policy and research priorities regarding the economic aspects of delivering primary care services. Many of the findings from the reviews have important implications for policymakers seeking to provide primary care services within the cost constraints that they face.

Our review contributes to health policy and health systems research in three ways. First, whereas earlier reviews of the economics of primary health care focused on specific geographic areas or study methods, our review is a comprehensive analysis of a large number of reviews that is not limited to high‐, middle‐, or low‐income countries or to specific study methodologies.^18^, ^19^ Second, our robust evaluation of both the conclusions and the quality of research included in our review allowed us to contribute a well‐grounded discussion and recommendations. Third, we built on the earlier literature to develop a definition and taxonomy to classify evidence regarding the economic aspects of delivering primary care services.^20^

We identified several important findings. For coverage arrangements, we found evidence that in some circumstances, reimbursement restriction policies and generic drug policies can be cost‐effective strategies.^57^ For financing arrangements, we found evidence that blended capitation payments and pay‐for‐performance payment mechanisms may reduce costs.^58^, ^59^, ^62^, ^63^, ^64^, ^65^ For service delivery arrangements, we found evidence that health workers' task shifting, telemedicine, computerized decision support systems, and patients' access to electronic health records can help reduce spending requirements.^67^, ^68^, ^78^, ^79^, ^83^, ^84^, ^85^, ^87^, ^88^, ^89^, ^90^, ^91^, ^92^ For governance arrangements, we found limited evidence that targets, clinical guidelines, and antimicrobial stewardship programs may have positive economic impacts overall.^106^, ^107^, ^108^

We should point out that the economic findings we highlighted from our included reviews, as well as those found elsewhere on these same topics, can form only a part of policy formulation and implementation, including individuals' access to care and the quality of care they receive, both of which have long‐term economic implications for health systems. This principle is the same whether the evidence suggests that an intervention or policy will have positive, negligible, or negative impacts or that no clear findings at all can be drawn. Economic findings, like those presented here, may also have different implications when implemented at different scales, such as the differences between local application and national rollout. Nevertheless, the large number and broad range of included reviews indicate the potential value of having a broad knowledge of different economic aspects of delivering primary care services and of considering the available findings in policy formulation processes.

### Limitations of Our Review

We applied a transparent, rigorous, and pragmatic approach to this evidence synthesis, compiling a more comprehensive and up‐to‐date review of evidence than previous reviews. Nonetheless, we dealt with similar challenges and limitations like those noted by the authors of other reviews of reviews. These included the scope of the evidence base and the quality of studies in the included reviews. For example, we relied on the quality and comprehensiveness of English‐language systematic reviews and meta‐analyses.^37^ With more time and resources, it may have been possible to use a framework like GRADE to assess the quality of each study identified by included reviews, or to apply guidance like PROGRESS to assess equity implications of the evidence in included systematic reviews.^194^, ^195^ Our inability to do so within the bounds of this exercise meant that instead of independently determining the relative strength or weakness of the available evidence, we had to consider the strengths and weaknesses of the included reviews and share the evidence as it was shared in those reviews in as standard a manner as possible. Future efforts could build on the use of these approaches in other reviews of reviews.^56^, ^196^

To ensure feasibility, we required that a systematic review be eligible for inclusion only if did not place eligibility restrictions on primary research that were based on the study participants' characteristics. This in turn meant excluding candidate reviews focused on specific socioeconomic, demographic, or health‐related groups. Nonetheless, the authors of a previous review similar to this one noted that the studies included in population‐specific systematic reviews will often be captured by other systematic reviews with broader population inclusion criteria.^18^ We also acknowledge that our review is limited by concentrating on the economic aspects of primary care services, and so we could not thoroughly explore other important facets of delivering primary care services, such as individuals' access to, and the quality of, those services. In addition, we accept that even though an umbrella review requires a disciplined research process, it cannot be described as entirely objective. As a result, the findings presented here should be considered in the context of the authors' interpretation of findings as they were abstracted, assessed for quality, and synthesized. We hope this review offers a platform to engage with the economic aspects of key population‐specific issues, such as the delivery of primary care services to treat chronic diseases or the progress toward achieving universal access to quality primary care services in communities and society.

### Implications for Future Research

We identified several topics ripe for further research to support informed policymaking on the economic aspects of delivering primary care services. Supplementary File 3 outlines many of these areas in further detail; we have discussed only a sample here. For coverage arrangements, there are opportunities to conduct systematic reviews focused on the economic aspects of ensuring that primary care services are accessible and comprehensive and to undertake primary research on the use of preferred drugs lists and interventions to shift health care use to general primary care settings. Future research on financing arrangements could include systematic reviews of the economic aspects of mobilizing and disbursing funding for primary care services and facilities, and primary research on the direct and unintended consequences of using financial incentives in primary care services. Future research on service delivery arrangements could include systematic reviews of the economic aspects of management of primary care professionals' various responsibilities and to primary research on the organization of primary care infrastructure. Finally, systematic reviews of governance arrangements in low‐income countries and research on patients' engagement in clinical decision making in primary care settings are needed.

Although we identified and synthesized many systematic reviews and reviews of reviews focused on topics related to economic aspects of delivering primary care services, the subject area represents a rather small segment of syntheses of evidence in research for health. Directing more resources to the greater creation, dissemination, and utilization of research on the economic aspects of delivering primary care services will be critical to achieve universal health coverage and to meet evolving population health needs. In addition, more effort is needed to place this evidence in the context of the trade‐offs faced by policymakers in their pursuit of supplying fair, safe, and sustainable primary care services, as well as the complex web of factors, such as socioeconomic and commercial determinants, that influence population health outcomes.^197^

## Conclusions

This evidence synthesis offers a platform to inform policy and research priorities regarding the economic aspects of delivering primary care services. Similar to previous reviews on this topic, we highlight that more relevant and high‐quality research and evidence are required to support the rhetoric calling for greater investments in primary health care. Generating high‐quality and context‐relevant evidence is a challenging, but achievable, goal. With increased investments, future efforts could include the development of a road map for research on the economics of delivering primary care services, or a guide to support researchers and policymakers trying to evaluate their policies on a routine and ad hoc basis. Taking action will require sustained and coordinated support for research by the funders, providers, and users of health research.^18^, ^198^ We hope that the findings of this review can help advocate for and direct that action.

1

*Funding/Support*: Funding to support this work was provided by the WHO Regional Office for Europe (WHO/Europe) and LSE Health.

*Conflict of Interest Disclosures*: All authors completed the ICMJE Form for Disclosure of Potential Conflicts of Interest. No conflicts were reported.

*Acknowledgments*: Lorcan Clarke, Michael Anderson, Rob Anderson, Morten Bonde Klausen, Søren Rud Kristensen, Pavlos Theodorakis, Jose Valderas, and Elias Mossialos designed the study and developed and refined the study protocol. Lorcan Clarke and Morten Bonde Klausen conducted the title and abstract screening, with Rob Anderson and Michael Anderson acting as third reviewers. Lorcan Clarke, Jenna Kerns, and Adrian Rabe conducted the full text screening, with Rob Anderson acting as a third reviewer. Lorcan Clarke and Michael Anderson conducted the quality assessment, and Lorcan Clarke and Rebecca Forman conducted the data abstraction. Lorcan Clarke completed the data analysis and initial drafting of the manuscript, and Lorcan Clarke, Michael Anderson, Rebecca Forman, and Elias Mossialos made the final changes. Morten Bonde Klausen, Rob Anderson, Adrian Rabe, Søren Rud Kristensen, Pavlos Theodorakis, Jose Valderas, and Hans Kluge contributed to refining the manuscript. All of the authors approved the manuscript.

## Supporting information

Supplementary File 1. Methods

Supplementary File 2. Publications

Supplementary File 3. Findings
