# Supplementary material for: Economic Aspects of Delivering Primary Care Services: An Evidence Synthesis to Inform Policy and Research Priorities
Source: Milbank Q. 2021 Sep 2;99(4):974–1023. doi: 10.1111/1468-0009.12536 (PMC8718591; doi:10.1111/1468-0009.12536)
Supplement: Supplementary file 1 — Methods [file MILQ-99-974-s003.docx]

# Economic Aspects of Delivering Primary Care Services – An Evidence Synthesis to Inform Policy and Research Priorities (Supplementary File 1 – Methods)

This file summaries our search strategy, data abstraction, and quality assessment processes.

# Search Strategy

## EMBASE (Ovid) and Medline (Ovid)

1. ("allied health" adj (professional* or personnel or worker* or "support worker*")).ab.ti
2. (paramedic* or paraprofessional* or (ambula* adj (health* or care)).ab.ti
3. (community adj (care* or "drug distributor*" or nurs* or pharmac* or medic* or "mental health") or (community adj (health or healthcare) adj (volunteer* or worker* or agent* or guide* or visitor* or advocate* or promoter* or carer* or aide* or support* or assistant* or service*)) or ("health volunteer" or "treatment supporter")).ab.ti
4. ("General practi*" or "emergenc*" or (home adj (visit* or health* or care)) or ((integrated or shared) adj (care or health*)) or (rural adj (care or health*))).ab.ti
5. (GP* or (family adj (Physic* or medic* or doctor*))).ab.ti
6. (primary adj (health* or care)).ab.ti
7. (nurse* or midwi* or birth attendant*).ab.ti
8. (Pharmacy or pharmacist or pharmacies).ab.ti
9. ((preventive adj (care or health* or service* or program*)) or (promot* adj (health* or care))).ab.ti
10. (telemedicine or "mobile health" or mHealth or telehealth or eHealth or "remote consultation" or teleconsult* or telehealthcare or telemonitoring or "remote monitoring" or tele$education or "health messages" or "educational technology" or "decision support system" or "remote diagnosis" or telediagnosis or videoconferencing or telemanagement or tele$management or teleconsult).ab.ti
11. (local adj (clinic* or hospital* or "health cent*" or "health facility*"))).ab.ti
12. 1 or 2 or 3 or 4 or 5 or 6 or 7 or 8 or 9 or 10 or 11
13. ("*admission*" or cost* or budget* or contract* or efficien* or financ* or fiscal* or fund* or generic or hospitali$ation* or invest* or pharmacoeconomic* or "*prescrib*" or prescript* or pric* or procur* or refer* or replac* or resilience or resource* or retent* or spend* or socioeconomic* or substitut* or supply or turnover).ab.ti
14. (charge* or expen* or fee* or pay* or time).ab.ti
15. (econom* or "*equal*" or "*equit*"))).ab,ti.
16. 13 or 14 or 15
17. ("cochrane review" or "critical review" or "evidence gap map" or "integrative review" or "mapping review" or "meta-analysis" or "meta analysis" or "mixed studies review" or "mixed methods review" or "qualitative evidence synthesis" or "rapid review" or "realist review" or "scoping review" or "state-of-the-art review" or "systematic evaluation" or "systematic literature review" or "systematic review" or "systematic search and review" or "systematized review" or "umbrella review").ab.ti
18. 12 and 16 and 17
19. Limit 18 to (embase or medline)

## Cochrane Library

- (charge* OR expen* OR fee* OR pay* OR time OR "*admission*" OR cost* OR budget* OR contract* OR efficien* OR financ* OR fiscal* OR fund* OR generic OR hospitali$ation* OR invest* OR pharmacoeconomic* OR "*prescrib*" OR prescript* OR pric* OR procur* OR refer* OR replac* OR resilience OR resource* OR retent* OR spend* OR socioeconomic* OR substitut* OR supply OR turnover OR econom* OR "*equal*" OR "*equit*" )
- **AND** ( ( "allied health" AND ( professional* OR personnel OR worker* OR "support worker*" ) ) OR paramedic* OR paraprofessional* OR ( ambula* AND ( health* OR care ) ) OR ( ( community AND ( care* OR "drug distributor*" OR nurs* OR pharmac* OR medic* OR "mental health" ) ) OR ( community AND ( health OR healthcare ) AND ( volunteer* OR worker* OR agent* OR guide* OR visitor* OR advocate* OR promoter* OR carer* OR aide* OR support* OR assistant* OR service* ) ) OR ( "health volunteer" OR "treatment supporter" ) ) OR ( "general practi*" OR gp* OR ( "emergenc*" ) OR ( home AND ( visit* OR health* OR care ) ) OR ( ( integrated OR shared ) AND ( care OR health* ) ) OR ( rural AND ( care OR health* ) ) OR ( specialist* ) ) OR family AND ( physic* OR medic* OR doctor* ) OR ( primary AND ( health* OR care ) ) OR ( nurse* OR midwi* OR birth AND attendant* ) OR ( pharmacy OR pharmacist OR pharmacies ) OR ( preventive AND ( care OR health* OR service* OR program* ) ) OR ( promot* AND ( health* OR care ) ) OR ( telemedicine OR "mobile health" OR mhealth OR telehealth OR ehealth OR "remote consultation" OR teleconsult* OR telehealthcare OR telemonitoring OR "remote monitoring" OR tele$education OR "health messages" OR "educational technology" OR "decision support system" OR "remote diagnosis" OR telediagnosis OR videoconferencing OR telemanagement OR tele$management OR teleconsult ) OR ( local AND ( clinic* OR hospital* OR "health cent*" OR "health facility*" ) ) )

## EconLit

- (("*admission*" or cost* or budget* or contract* or "*efficien*" or financ* or fiscal* or fund* or generic* or hospitali?ation* or invest* or pharmacoeconomic* or "*prescrib*" or prescript* or pric* or procur* or refer* or replac* or resilience or resource* or retent* or spend* or socioeconomic* or substitut* or supply or turnover) OR (charge* or expen* or fee* or pay* or time) OR (econom* or "*equal*" or "*equit*"))
- **AND** (("allied health" AND (professional* or personnel or worker* or "support worker*")) OR (paramedic* or paraprofessional* or (ambula* adj (health* or care))) OR ((community adj (care* or "drug distributor*" or nurs* or pharmac* or medic* or "mental health")) or (community adj (health or healthcare) AND (volunteer* or worker* or agent* or guide* or visitor* or advocate* or promoter* or carer* or aide* or support* or assistant* or service*)) or ("health volunteer" or "treatment supporter")) OR ("General practi*" or "emergenc*" or (home AND (visit* or health* or care)) or ((integrated or shared) AND (care or health*)) or (rural AND (care or health*))) OR (GP* or (family AND (Physic* or medic* or doctor*))) OR (primary AND (health* or care)) OR (nurs* or midwi* or birth attendant*) OR (Pharmacy or pharmacist or pharmacies) OR (preventive AND (care or health* or service* or program*)) OR (promot* AND (health* or care)) OR (telemedicine or "mobile health" or mHealth or telehealth or eHealth or "remote consultation" or teleconsult* or telehealthcare or telemonitoring or "remote monitoring" or tele?education or "health messages" or "educational technology" or "decision support system" or "remote diagnosis" or telediagnosis or videoconferencing or telemanagement or tele?management or teleconsult) OR (local AND (clinic* or hospital* or "health cent*" or "health facility*")))
- **AND** (("systematic review" or "meta?analys?s" or "cochrane review") OR ("mapping review" or "rapid review" or "scoping review" or "systematic search and review" or "umbrella review" or "overview of reviews" or "evidence gap map" or "systematic literature review") OR "realist review")

# Data Abstraction

| **Question** | ***Response*** | ***-*** | ***Further Comment*** |
| --- | --- | --- | --- |
| Review: | *x* | *x* | *x* |
| Extracted by: | *x* | *x* | *x* |
| Date: | *x* | *x* | *x* |
| Review objective/study question: | *x* | *x* | *x* |
| Date of last search: | *x* | *x* | *x* |
| **Search** | ***What the review authors searched for*** | ***What the review authors found*** | ***Further Comment*** |
| Study Designs | *x* | *x* | *x* |
| Interventions and Comparators | *x* | *x* | *x* |
| Participants | *x* | *x* | *x* |
| Outcomes | *x* | *x* | *x* |
| Settings | *x* | *x* | *x* |
| ***Results*** | ***Response*** | ***-*** | ***Further Comment*** |
| Findings | *x* | - | *x* |
| Limitations | *x* | - | *x* |
| Conclusions/Key Messages | *x* | - | *x* |
| List of included studies (citations) | *x* | - | *x* |

# Quality Assessment

The below table displays questions, criteria, and guidance used in quality assessment. These were separated into “Section A”, which focused on review methods related to identification of studies for inclusion, and “Section B”, which focused on review methods to analyse study findings.

Two authors drew upon previous uses of the tool to independently evaluate whether a review had “minor limitations”, “important limitations”, “fatal flaws” in Section A and Section B separately. The overall decisions of each review was based as follows: i) if both Section A and Section B had “minor limitations” then the review had “minor limitations”, ii) if one of Section A or Section B had “important limitations” then the review had “important limitations”, iii) if one of Section A or Section B had “fatal flaws” then the review had “fatal flaws”.

Examples of characteristics which led to “Important Limitations” included: review searches being conducted more than ten years prior to March 2019 (Section A), review not including extraction of data by ≥2 reviewers (Section B). Examples of characteristics which led to “Fatal Flaws” characterisation include a review not conducting a risk of bias assessment (Section A) or not including a table/summary of included studies (Section B).

Table 2: SUPPORT Tool used in Quality Assessment (Template)

| **Question** | ? | **Decision criteria** | **?** | **Decision guidance** | **Further Comment** |
| --- | --- | --- | --- | --- | --- |
| Review: |  | *.* |  |  |  |
| Assessed by: |  | *.* |  |  |  |
| Date: |  | *.* |  |  |  |
| A.1 Were the criteria used for deciding which studies to include in the review reported? | *.* | Types of studies | *.* | Yes: All 4 criteria met Can't tell/partially: 1-3 criteria met  No: 0 criteria met |  |
|  | *.* | Participants |  |  |  |
|  | *.* | Intervention(s) |  |  |  |
|  | *.* | Outcome(s) |  |  |  |
| A.2 Was the search for evidence reasonably comprehensive? | *.* | Language bias avoided  (No restriction of inclusion based on language) | *.* | Yes: All 5 criteria met Can't tell/partially: "Relevant databases" and "reference lists" criteria met No: "Relevant databases" and "reference lists" criteria NOT met |  |
|  | *.* | No restriction of inclusion based on publication status (i.e. whether literature is academic publication, grey literature or unpublished studies - e.g. working papers) |  |  |  |
|  | *.* | Relevant databases searched  (e.g. include, but not limited to, Medline + Cochrane Library) |  |  |  |
|  | *.* | Reference lists in included articles checked (may be referred to as "Snowball sampling") |  |  |  |
|  | *.* | Authors/experts contacted |  |  |  |
| A.3 Is the review reasonably up‐to‐date? | *.* | Were the searches done recently enough that more recent research is unlikely to be found or to change the results of the review? (Were the searches conducted in the past ten years?) | *.* | Can't tell/partially: Search dates not identified. |  |
| A.4 Was bias in the selection of articles avoided? | *.* | Explicit selection criteria | *.* | Yes: All 4 criteria met Can't tell/partially: 1-3 criteria met  No: 0 criteria met |  |
|  | *.* | Independent screening of full text by ≥ 2 reviewers |  |  |  |
|  | *.* | List of included studies provided |  |  |  |
|  | *.* | List of excluded studies provided |  |  |  |
| A.5 Did the authors use appropriate criteria to assess the risk for bias in analysing the studies that are included? | *.* | The criteria used for assessing the risk of bias were reported | *.* | Yes: All 3 criteria met Can't tell/partially: 1-2 criteria met  No: 0 criteria met |  |
|  | *.* | A table or summary of the assessment of each included study for each criterion was reported |  |  |  |
|  | *.* | Sensible criteria were used that focus on the risk of bias (and not other qualities of the studies, such as precision or applicability) |  |  |  |
| A.6 Overall – how would you rate the methods used to identify, include and critically appraise studies? | *.* | Number of Decisions reported as "Yes" | *.* | Reliable: Only minor limitations  Important: Limitations that are important enough that it would be worthwhile to search for another systematic review and to interpret the results of this review cautiously, if a better review cannot be found. If the "No" or "Partial" option is used for any of the questions above, the review is likely to have important limitations.  Major limitations: Limitations that are important enough that the results of the review are not reliable and they should not be used in the policy brief. Examples of major limitations might include not reporting explicit selection criteria, not providing a list of included studies or not assessing the risk of bias in included studies. |  |
|  | *.* | Number of Decisions reported as "Can't tell/partially" |  |  |  |
|  | *.* | Number of decisions reported as "No" |  |  |  |
| **Section B: Methods used to analyse the findings** |  |  |  |  |  |
| B.1 Were the characteristics and results of the included studies reliably reported? | *.* | ≥ 2 reviewers conduct data extraction | *.* | Yes: All 3 criteria met  Can't tell/partially: 1-2 criteria met   No: 0 criteria met  Not applicable: E.g. - No included studies |  |
|  | *.* | Table/summary provided: Characteristics of the participants, interventions and outcomes for the included studies |  |  |  |
|  | *.* | A table or summary (of study characteristics/conclusions) |  |  |  |
| B.2 Were the methods used by the review authors to analyse the findings of the included studies reported? |  |  | *.* | Please note synthesis approach |  |
| B.3 Did the review describe the extent of heterogeneity? | *.* | Did the review ensure that included studies were similar enough that it made sense to combine them, sensibly divide the included studies into homogeneous groups, or sensibly conclude that it did not make sense to combine or group the included studies? | *.* |  |  |
|  | *.* | Did the review discuss the extent to which there were important differences in the results of the included studies? |  |  |  |
|  | *.* | If a meta‐analysis was done, was the I2, chi square test for heterogeneity or other appropriate statistic reported? |  |  |  |
| B.4 Were the findings of the relevant studies combined (or not combined) appropriately relative to the primary question the review addresses and the available data? | *.* | Descriptive analysis only | *.* | Yes: If appropriate table, graph or meta‐analysis AND appropriate weights AND the extent of heterogeneity was taken into account, the answer is likely YES.  Can't tell/partially: If unsure: CAN'T TELL/PARTIALLY  No: If narrative OR vote counting (where quantitative analyses would have been possible) OR inappropriate table, graph OR meta‐analyses OR unit of analyses errors not addressed (and should have been) the answer is likely NO.  Not applicable: If no studies/no data: NOT APPLICABLE |  |
|  | *.* | Vote counting based on direction of effect |  |  |  |
|  | *.* | Vote counting based on statistical significance |  |  |  |
|  | *.* | Description of range of effect sizes |  |  |  |
|  | *.* | Meta‐analysis |  |  |  |
|  | *.* | Meta‐regression |  |  |  |
|  | *.* | Other: specify |  |  |  |
|  | *.* | Not applicable (e.g. no studies or no data) |  |  |  |
|  | *.* | ***If meta-analysis/meta-regression used:  How were the studies weighted in the analysis?*** |  |  |  |
|  | *.* | Equal weights (this is what is done when vote counting is used) |  |  |  |
|  | *.* | By quality or study design (this is rarely done) |  |  |  |
|  | *.* | Inverse variance (this is what is typically done in a meta‐analysis) |  |  |  |
|  | *.* | Number of participants |  |  |  |
|  | *.* | Other, specify: |  |  |  |
|  | *.* | Not clear |  |  |  |
|  | *.* | Not applicable (e.g. no studies or no data) |  |  |  |
|  | *.* | ***If meta-analysis/meta-regression used:  Did the review address unit of analysis errors?*** |  |  |  |
|  | *.* | Yes ‐ took clustering into account in the analysis (e.g. used intra‐cluster correlation coefficient) |  |  |  |
|  | *.* | No, but acknowledged problem of unit of analysis errors |  |  |  |
|  | *.* | No mention of issue |  |  |  |
|  | *.* | Not applicable ‐ no clustered trials or studies included |  |  |  |
| B.5 Did the review examine the extent to which specific factors might explain differences in the results of the included studies?` | *.* | Were factors that the review authors considered as likely explanatory factors clearly described? | *.* | Yes: Both explanatory factors and heterogeneity considered.   Can't tell/partially: Only one of explanatory factors and heterogeneity considered.   No: Niether considered.   Not applicable: e.g. too few studies, no important differences in the results of the included studies, or the included studies were so dissimilar that it would not make sense to explore heterogeneity of the results |  |
|  | *.* | ***Was a sensible method used to explore the extent to which key factors explained heterogeneity? (Sensible can be any of below options which clearly involve exploration of heterogeneity)*** |  |  |  |
|  | *.* | Descriptive/textual |  |  |  |
|  | *.* | Graphical |  |  |  |
|  | *.* | Meta‐regression |  |  |  |
|  | *.* | Other |  |  |  |
| B.6 Overall ‐ how would you rate the methods used to analyse the findings relative to the primary question addressed in the review? | *.* | Number of Decisions reported as "Yes" | *.* | Reliable (only minor limitations): Summary assessment score B relates to the 5 questions in this section, regarding the analysis.  Important limitations - Limitations that are important enough that it would be worthwhile to search for another systematic review and to interpret the results of this review cautiously, if a better review cannot be found. If the “No” or ”Partial” option is used for any of the 5 preceding questions, the review is likely to have important limitations.  Fatal flaws - Limitations that are important enough that the results of the review are not reliable and they should not be used in the policy brief. Examples of major limitations might include not reporting critical characteristics of the included studies or not reporting the results of the included studies. |  |
|  | *.* | Number of Decisions reported as "Can't tell/partially" |  |  |  |
|  | *.* | Number of decisions reported as "No" |  |  |  |
|  | *.* | Number of decisions reported as "Not applicable" |  |  |  |
| **Section C: Overall assessment of the reliability of the review** |  |  |  |  |  |
| C.1 Are there any other aspects of the review not mentioned before which lead you to question the results? | *.* | Additional methodological concerns |  |  | Add reasons/comments here |
|  | *.* | Conflicts of interest (of the review authors or for included studies) |  |  |  |
|  | *.* | Interpretation |  |  |  |
|  | *.* | No other quality issues identified |  |  |  |
|  | *.* | Other |  |  |  |
|  | *.* | Robustness |  |  |  |
| C.2 Based on the above assessments of the methods how would you rate the reliability of the review | *.* | ***Response to A: A.6 Overall – how would you rate the methods used to identify, include and critically appraise studies?*** | *.* |  | Add reasons/comments here |
|  | *.* | ***Response to B: B.6 Overall ‐ how would you rate the methods used to analyse the findings relative to the primary question addressed in the review?*** |  |  |  |

Source: Adapted from SUPPORT Tools for evidence-informed health policymaking^1,2^ and approaches used in similar broad-focused umbrella reviews.^3–6^

# Study Protocol

This protocol is registered under the following citation:

- Lorcan Clarke, Michael Anderson, Elias Mossialos, Søren Rud Kristensen, Jose Valderas, Rob Anderson, Pavlos Theodorakis, Hans Kluge, Helene Probst, Morten Sall Jensen, Morten Bonde Klausen. The economics of delivering primary care services: an overview of the evidence. PROSPERO 2019 CRD42019125040 Available from: <https://www.crd.york.ac.uk/prospero/display_record.php?ID=CRD42019125040>

## Review question

This review will identify, assess, and synthesise evidence on the economics of delivering primary care services. We shall:

• Define “the economics of delivering primary care services”.

• Identify, describe, and assess published evidence reviews.

• Analyse the economic evidence on across different components of primary care services.

• Synthesise conclusions for stakeholder review and create a platform for further research.

## Searches

Sources:

• Bibliographic databases will include: the Cochrane Library (EPOC), EconLit, EMBASE (Ovid) and MEDLINE (Ovid).

• Repositories of grey literature include: OECD iLibrary, WHOLIS and the World Bank eLibrary.

• Additional sources will include:

- The reference lists of reviews included for evidence synthesis.

- Reviews identified by evidence gap maps, mapping reviews, overviews of reviews, rapid reviews, realist reviews, scoping reviews, systematic search and reviews, and umbrella reviews which appear in our study searches.

- Submission of papers by members of the Economic Taskforce on Primary Health Care.

Search dates:

We include reviews published from 1 January 1978, the year of the Alma-Ata Declaration, to 4 March 2019.

Restrictions:

We will place no restrictions on the settings of studies in included reviews, or the scope of included reviews. We will limit inclusion to full-text English language reviews.

Additional search strategy information can be found in the attached PDF document (link provided below).

## Types of study to be included

We will limit included reviews to evidence reviews which synthesise evidence on the economics of delivering primary care services, and which have a clear search and inclusion strategy and clear methods for assessing and synthesising evidence.

We will only synthesise our results from the following types of evidence reviews: Cochrane reviews, meta-analyses, and systematic reviews.

We will use the following types of evidence reviews for identifying additional reviews and informing our discussions, these include: evidence gap maps, mapping reviews, overviews of reviews, rapid reviews, scoping reviews, realist reviews, and umbrella reviews.

We will not consider reviews which evaluate studies only looking at health outcomes. Health outcomes must be linked to quantifiable changes in future health costs, future incomes or future care admissions would suffice.

We will not consider the economics of the broader societal impacts of delivering primary care services. These include labour markets, productivity, economic growth, and social cohesion. This due to feasibility reasons, our intended audience, and to allow a future review, or review of reviews, to specifically focus on that topic alone.

## Condition or domain being studied

The delivery of primary care services.

## Participants/population

No restrictions will be imposed on the participants of the studies in included evidence reviews.

We will include studies in which reviews are the unit of inclusion, rather than people/study participants.

## Intervention(s), exposure(s)

This review is restricted to evidence reviews that evaluate studies of interventions or approaches connected to “delivering primary care services”.

We define “Delivering primary care services” as "Arrangements and resources directly supporting the functioning and implementation of primary care services. Primary care services are multidisciplinary quality health care services that support the core system´s functions of first-contact, continuity, comprehensiveness (including promotive, protective, preventive, curative, rehabilitative, and palliative care), and coordination."
We define "The economics of delivering primary care services" as follows: “Quantifiable outcomes on current or future resources available to users and providers attributed to arrangements and resources directly supporting the functioning and implementation of primary care services. Primary care services are multidisciplinary health care services that support the core system´s functions of first-contact, continuity, comprehensiveness (including promotive, protective, preventive, curative, rehabilitative, and palliative care), and coordination."

## Comparator(s)/control

This review will limit inclusion to evidence reviews which synthesis conclusions across multiple studies that compare the outcomes of interventions versus control groups which receive no intervention, another intervention, or current practice.

## Main outcome(s)

To provide an overview of the evidence on the economics of delivering primary care services.

## Additional outcome(s)

None.

## Data extraction (selection and coding)

Two independent reviewers will screen titles and abstracts of all evidence reviews. Full texts of candidates for inclusion will be assessed by two independent reviewers and reasons for exclusion at that stage of the screening process will be reported. All screening activities will use the eligibility criteria set out in the eligibility criteria.

We will extract information from reviews for our results synthesis into an Excel spreadsheet. At present, there is no consolidated guidance on for extraction of data items for reviews of reviews. We will develop and share our data extraction criteria, which will be informed by criteria recommended by: AMSTAR 2, PROGRESS and available guidance from peer-reviewed publications on overviews of reviews, as well as additional approaches used in analysis of primary research for health (e.g. CHEERS checklist, iDSI reference case).

## Risk of bias (quality) assessment

Two independent reviewers will assess the quality of included reviews using guidance from available tools. We shall assess and incorporate guidance from the AMSTAR 2 tool and/or the ROBIS tool. Any disagreements will be recorded and noted where appropriate in the final publication. Results will be reported in summary tables.

Two independent reviewers will assess the quality of evidence within reviews using the GRADE tool. The GRADE tool was developed specifically for judgements of quality of evidence during guideline development, and also adopted for judgement of quality of evidence within Cochrane reviews. Any disagreements will be recorded and noted where appropriate in the final publication. Results will be reported in summary tables.

## Strategy for data synthesis

Data synthesis will involve sorting and analysing included reviews according to evidence types and evidence topics. We will use available guidance to address methodological challenges in reviews of reviews. Structured summary tables will be provided as is appropriate.

We anticipate that there will be limited scope for meta-analysis because of the range of interventions and settings involved included reviews.

## Analysis of subgroups or subsets

We expect to structure our synthesis around the outcome focus of reviews (i.e. users or providers of primary care services) and the components (i.e. financing, governance, information and technology, infrastructure, and workforce) or implementation strategies involved in delivery of primary care services.

## Contact details for further information

Lorcan Clarke

[l.clarke3@lse.ac.uk](mailto:l.clarke3@lse.ac.uk)

## Organisational affiliation of the review

London School of Economics and Political Science (LSE), Imperial College London (ICL), Danish National Centre for Social Research (VIVE), University of Exeter (UoE), World Health Organization Regional Office for Europe (WHO/Europe), Danish Health Authority (SST).

## Review team members and their organisational affiliations

Mr Lorcan Clarke. London School of Economics and Political Science
Dr Michael Anderson. London School of Economics and Political Science
Professor Elias Mossialos. London School of Economics and Political Science; Imperial College London
Dr Søren Rud Kristensen. Imperial College London
Professor Jose Valderas. University of Exeter
Professor Rob Anderson. University of Exeter
Dr Pavlos Theodorakis. World Health Organization Regional Office for Europe (WHO/Europe)
Dr Hans Kluge. World Health Organization Regional Office for Europe (WHO/Europe)
Miss Helene Probst. Danish Health Authority (SST)
Dr Morten Sall Jensen. Danish National Centre for Social Research (VIVE)
Dr Morten Bonde Klausen. Danish National Centre for Social Research (VIVE)

## Collaborators

WHO/Europe Economic Taskforce on Primary Health Care .

## Type and method of review

Cost effectiveness, Review of reviews, Service delivery, Systematic review

## Anticipated or actual start date

08 March 2019

## Anticipated completion date

31 July 2019

## Funding sources/sponsors

World Health Organization Regional Office for Europe (WHO/Europe); Danish Health Authority (SST)

## Conflicts of interest

None known

## Language

English

## Country

Ireland

## Stage of review

Review Ongoing

## Subject index terms status

Subject indexing assigned by CRD

## Subject index terms

Delivery of Health Care; Health Care Costs; Humans; Primary Health Care; Public Health

## Date of registration in PROSPERO

10 April 2019

## Date of first submission

08 March 2019

## Stage of review at time of this submission

| **Stage** | **Started** | **Completed** |
| --- | --- | --- |
| Preliminary searches | Yes | No |
| Piloting of the study selection process | No | No |
| Formal screening of search results against eligibility criteria | No | No |
| Data extraction | No | No |
| Risk of bias (quality) assessment | No | No |
| Data analysis | No | No |

*The record owner confirms that the information they have supplied for this submission is accurate and complete and they understand that deliberate provision of inaccurate information or omission of data may be construed as scientific misconduct.*

*The record owner confirms that they will update the status of the review when it is completed and will add publication details in due course.*

# References

1 Lavis JN, Oxman AD, Souza NM, Lewin S, Gruen RL, Fretheim A. SUPPORT Tools for evidence-informed health Policymaking (STP) 9: Assessing the applicability of the findings of a systematic review. *Heal Res Policy Syst* 2009; **7**: S9.

2 Lewin S, Oxman AD, Lavis JN, Fretheim A. SUPPORT Tools for evidence-informed health Policymaking (STP) 8: Deciding how much confidence to place in a systematic review. *Heal Res Policy Syst* 2009; **7**: S8.

3 Herrera CA, Lewin S, Paulsen E, *et al.* Governance arrangements for health systems in low-income countries: An overview of systematic reviews. Cochrane Database Syst. Rev. 2017; **2017**. DOI:10.1002/14651858.CD011085.pub2.

4 Ciapponi A, Lewin S, Herrera CA, *et al.* Delivery arrangements for health systems in low-income countries: An overview of systematic reviews. Cochrane Database Syst. Rev. 2017; **2017**. DOI:10.1002/14651858.CD011083.pub2.

5 Wiysonge CS, Paulsen E, Lewin S, *et al.* Financial arrangements for health systems in low-income countries: An overview of systematic reviews. Cochrane Database Syst. Rev. 2017; **2017**. DOI:10.1002/14651858.CD011084.pub2.

6 Pantoja T, Opiyo N, Lewin S, *et al.* Implementation strategies for health systems in low-income countries: An overview of systematic reviews. Cochrane Database Syst. Rev. 2017; **2017**. DOI:10.1002/14651858.CD011086.pub2.
