# Supplementary material for: Economic Aspects of Delivering Primary Care Services: An Evidence Synthesis to Inform Policy and Research Priorities
Source: Milbank Q. 2021 Sep 2;99(4):974–1023. doi: 10.1111/1468-0009.12536 (PMC8718591; doi:10.1111/1468-0009.12536)
Supplement: Supplementary file 2 — Publications [file MILQ-99-974-s002.docx]

# Economic Aspects of Delivering Primary Care Services – An Evidence Synthesis to Inform Policy and Research Priorities (Supplementary File 2 - Publications)

This supplementary file provides a full list of included systematic reviews and lists reviews of reviews we used to inform our further analysis.

# Included reviews with minor/important limitations

| Review | Aim | Limitations |
| --- | --- | --- |
| Abdulwahid et al (2017) The impact of senior doctor assessment at triage on emergency department performance measures: systematic review and meta-analysis of comparative studies | To determine if placing a senior doctor at triage versus standard single nurse in a hospital emergency department (ED) improves ED performance. | Minor |
| Akbari et al (2008) Interventions to improve outpatient referrals from primary care to secondary care | To estimate the effectiveness and efficiency of interventions to change outpatient referral rates or improve outpatient referral appropriateness. | Important |
| Akiyama and Yoo (2017) A Systematic Review of the Economic Evaluation of Telemedicine in Japan | To assess published economic evaluations on telemedicine in Japan, focusing on economic efficiency of interventions, methodological rigour and research priorities. | Minor |
| Baker et al (2015) Tailored interventions to address determinants of practice | To determine whether tailored intervention strategies are effective in improving professional practice and healthcare outcomes. Compared interventions tailored to address the identified determinants of practice with either no intervention or interventions not tailored to the determinants. | Minor |
| Baxter et al (2018) The effects of integrated care: a systematic review of UK and international evidence | To assess the effects of integration or co-ordination between healthcare services, or between health and social care on service delivery outcomes including effectiveness, efficiency and quality of care. | Minor |
| Bennett and Glasziou (2003) Computerised reminders and feedback in medication management a systematic review of randomised controlled trials | To assess the benefits of computerised systems that support medication management through reminders or feedback to healthcare providers, or reminders to patients | Minor |
| Bickerdike et al (2017) Social prescribing: less rhetoric and more reality. A systematic review of the evidence. | To summarise the evidence for the effectiveness of social prescribing programmes relevant to the UK NHS setting. | Minor |
| Bosch-Capblanch et al (2011) Managerial supervision to improve primary health care in low- and middle-income countries. | To review the effects of managerial supervision of health workers on the quality of primary healthcare in low- and middle-income countries. | Minor |
| Brainard et al (2015) A systematic review of health service interventions to reduce use of unplanned health care in rural areas | To assess the effectiveness of interventions to reduce the use of unplanned healthcare by rural populations. | Important |
| Brocklehurst et al (2013) The effect of different methods of remuneration on the behaviour of primary care dentists | To evaluate the effects of different methods of remuneration on the level and mix of activities provided by primary care dentists and the impact this has on patient outcomes. | Minor |
| Bullard et al (2011) The role of a rapid assessment zone/pod on reducing overcrowding in emergency departments: a systematic review | To summarise the available evidence assessing the impact of rapid assessment zones/pods (RAZ/RAP) to mitigate emergency department (ED) overcrowding, and to evaluate the quality of the current research. | Minor |
| Bunn et al (2004) Telephone consultation and triage: effects on health care use and patient satisfaction | To assess the effects of telephone consultation on safety, service usage and patient satisfaction and to compare telephone consultation by different healthcare professionals. | Important |
| Cabilan and Boyde (2017) A systematic review of the impact of nurse-initiated medications in the emergency department | To review evaluations of the effects of nurse-initiated medications in the emergency department and to quantify the impact of the practice on quality of care indicators. | Minor |
| Carter et al (2016) The impact of primary care reform on health system performance in Canada: a systematic review | To synthesise evidence on Canadian primary care reforms. | Minor |
| Chan et al (2018) Clinical and economic outcomes of nurse-led services in the ambulatory care setting: A systematic review | To assess the clinical effectiveness of nurse-led services in the ambulatory or community care setting. Secondary aims include to examine the economic outcomes and to characterise the nurse-led services. | Important |
| Crawford et al (2017) The impact of walk-in centres and GP co-operatives on emergency department presentations: A systematic review of the literature | This systematic review aims to identify the impact of alternative emergency care pathways on ED presentations – specifically GP cooperatives and walk-in clinics. | Minor |
| de Barra et al (2018) Pharmacist services for non‐hospitalised patients | To examine the effect of pharmacists' non-dispensing services on non-hospitalised patient outcomes. | Important |
| de Bont et al (2015) Patient information leaflets to reduce antibiotic use and reconsultation rates in general practice: a systematic review | The aim of this systematic review is to study the effect of using patient information leaflets on antibiotic use and reconsultation rates in general practice consultations for common infections. | Minor |
| Doan et al (2011) A systematic review: The role and impact of the physician assistant in the emergency department | To assess the role of PAs in the ED, their impact on ED efficiency and on patient satisfaction | Important |
| Donald et al (2015) Hospital to community transitional care by nurse practitioners: A systematic review of cost-effectiveness | We summarise the results of RCTs evaluating the cost-effectiveness of nurse practitioners delivering transitional care in alternative or complementary roles and formulate recommendations based on the evidence | Minor |
| Downes et al (2017) Telephone consultations for general practice: a systematic review | The aim of this review is to utilise a systematic review to collate evidence on the use of telephone consultation as an alternative to face-to-face general practice visits. | Minor |
| Dudley and Garner (2011) Strategies for integrating primary health services in low- andmiddle-income countries at the point of delivery | To assess the effects of strategies to integrate primary healthcare services on healthcare delivery and health status in low- and middle-income countries. | Minor |
| Dyer et al (2014) Dental auxiliaries for dental care traditionally provided by dentists | To assess the effectiveness of dental auxiliaries in providing care traditionally provided by dentists. | Minor |
| Elrashidi et al (2018) Co-located specialty care within primary care practice settings: A systematic review and meta-analysis | To evaluate the impact of co-located specialty care services in primary care practice settings. | Minor |
| Estai et al (2017) A systematic review of the research evidence for the benefits of teledentistry | To present an overview of the evidence for the effectiveness and economic impact of teledentistry. | Important |
| Flodgren et al (2015) Interactive telemedicine: effects on professional practice and health care outcomes | To assess the effectiveness, acceptability and costs of interactive TM as an alternative to, or in addition to, usual care. | Minor |
| Flores-Mateo et al (2012) Effectiveness of Organizational Interventions to Reduce Emergency Department Utilization: A Systematic Review | To review the effectiveness of organisational interventions intended to reduce ED utilisation in the general population. | Minor |
| Flynn et al (2012) Engaging Patients in Health Care Decisions in the Emergency Department Through Shared Decision‐making: A Systematic Review | To assess approaches, methods, or tools to engage patients in SDM in the ED setting. | Minor |
| Fønhus et al (2018) Patient‐mediated interventions to improve professional practice | To assess the effectiveness of patient-mediated interventions on healthcare professionals’ performance. | Minor |
| Galipeau et al (2015) Effectiveness and Safety of Short‐stay Units in the Emergency Department: A Systematic Review | To evaluate the effectiveness and safety of ED short-stay units. | Minor |
| Gielen et al (2014) The effects of nurse prescribing: A systematic review | To assess the effects of nurse prescribing when compared to physician prescribing on the quantity and types of medication prescribed and on patient outcomes. | Minor |
| Gillam et al (2012) Pay-for-Performance in the United Kingdom: Impact of the Quality and Outcomes Framework—A Systematic Review | To review evidence on the Quality and Outcomes Framework (UK) on the quality of primary medical care. | Minor |
| Gonçalves‐Bradley et al (2018) Primary care professionals providing non‐urgent care in hospital emergency departments | To assess the effects of locating primary care professionals in hospital EDs to provide care for patients with non-urgent health problems. | Minor |
| Gosden et al (2000) Capitation, salary, fee‐for‐service and mixed systems of payment: effects on the behaviour of primary care physicians | To evaluate the impact of different methods of payment to primary care physicians. | Important |
| Green et al (2010) Pharmaceutical policies: effects of restrictions on reimbursement | To determine the effects of a pharmaceutical policy restricting the reimbursement of selected medications on drug use, healthcare utilisation, health outcomes and costs (expenditures). | Important |
| Gruen et al (2003) Specialist outreach clinics in primary care and rural hospital settings. | To review and assess the effectiveness of specialist outreach clinics. | Important |
| Halter et al (2013) The contribution of Physician Assistants in primary care: a systematic review | To assess evidence of the contribution of PAs within primary care, defined for this study as general practice, relevant to the UK or similar systems. | Important |
| Haroun et al (2016) The effects of interventions on quality of life, morbidity and consultation frequency in frequent attenders in primary care: A systematic review | To assess use and effects of interventions for frequent attenders in primary care. | Important |
| Hatah et al (2014) A systematic review and meta‐analysis of pharmacist‐led fee‐for‐services medication review | To the impact of fee-for-service pharmacist-led medication review on patient outcomes and quantify these according to the type of review undertaken. | Minor |
| Hedden et al (2014) The implications of the feminization of the primary care physician workforce on service supply: a systematic review | To examine evidence related to the effect of the increasing proportion of women working in primary care physician services. | Minor |
| Hone et al (2017) Does charging different user fees for primary and secondary care affect first-contacts with primary healthcare? A systematic review | To evaluate the impact of introducing differential user charges on service utilisation. | Minor |
| Houle et al (2012) Does performance-based remuneration for individual health care practitioners affect patient care?: a systematic review | To evaluate the effect of P4P remuneration targeting individual healthcare providers | Minor |
| Huntley et al (2014) Which features of primary care affect unscheduled secondary care use? A systematic review | To assess factors and interventions at primary care practice level that impact on levels of utilisation of unscheduled secondary care. | Important |
| Iribarren et al (2017) What is the economic evidence for mHealth? A systematic review of economic evaluations of mHealth solutions. | To assess evidence related to economic evaluations of mHealth interventions. | Minor |
| Irving et al (2017) International variations in primary care physician consultation time: a systematic review of 67 countries | To describe the average primary care physician consultation length in economically developed and low-income/middle-income countries, and to examine the relationship between consultation length and organisational-level economic, and health outcomes. | Minor |
| Ismail et al (2013) Reducing inappropriate accident and emergency department attendances: a systematic review of primary care service interventions | To review the evidence on primary care service interventions to reduce inappropriate A&E attendances. | Important |
| James et al (2018) Does Implementation of Biomathematical Models Mitigate Fatigue and Fatigue-related Risks in Emergency Medical Services Operations? A Systematic Review | To systematically review the evidence for the effectiveness of biomathematical models for fatigue mitigation. | Minor |
| Jennings et al (2015) The impact of nurse practitioner services on cost, quality of care, satisfaction and waiting times in the emergency department: A systematic review | To provide the best available evidence to determine the impact of nurse practitioner services on cost, quality of care, satisfaction and waiting times in the emergency department for adult patients. | Important |
| Johnson et al (2018) The impact of rural outreach programs on medical students’ future rural intentions and working locations: a systematic review | To examine the evidence base of rural educational programmes within medical education and focusses on workforce intentions and employment outcomes. | Minor |
| Kalankesh et al (2016) Effect of Telehealth Interventions on Hospitalization Indicators: A Systematic Review | To investigate the effect of telehealth interventions on hospitalisation rate and length of stay. | Minor |
| Kaufman et al (2017) Impact of Accountable Care Organizations on Utilization, Care, and Outcomes: A Systematic Review | To evaluate the evidence regarding the association of public and private ACOs with health service use, processes, and outcomes of care. | Minor |
| Kirkland et al (2018) A systematic review examining the impact of redirecting low-acuity patients seeking emergency department care is the juice worth the squeeze | To examine the impact of interventions designed to either bypass the ED or direct patients to other alternative care after ED presentation. | Minor |
| Kiwanuka et al (2011) Interventions to manage dual practice among health workers | To assess the effects of regulations implemented to manage dual practice. | Minor |
| Koehlmoos et al (2009) The effect of social franchising on access to and quality of health services in low‐ and middle‐income countries | To examine the evidence that social franchising has on access to and quality of health services in low- and middle-income countries. | Important |
| Krogsbøll et al (2019) General health checks in adults for reducing morbidity and mortality from disease | To quantify the benefits and harms of general health checks. | Minor |
| Laurant et al (2018) Nurses as substitutes for doctors in primary care | To investigate the impact of nurses working as substitutes for primary care doctors. | Minor |
| Leibowitz et al (2003) A systematic review of the effect of different models of after-hours primary medical care services on clinical outcome, medical workload, and patient and GP satisfaction. | To determine what evidence exists about the effect of different models of out-of-hours primary medical care service on outcome. | Important |
| Lidal et al (2013) Triage systems for pre-hospital emergency medical services - a systematic review | To evaluate the effect of validated triage systems for use in the pre-hospital setting. | Minor |
| Losier et al (2017) A Systematic Review of Antimicrobial Stewardship Interventions in the Emergency Department. | To characterise antimicrobial stewardship (AMS) in the ED and to identify interventions that improve patient outcomes or process of care and/or reduce consequences of antimicrobial use. | Important |
| Maillet et al (2018) Laboratory testing in primary care: A systematic review of health IT impacts | To synthesise impacts resulting from the use of health information technology in each phase of the laboratory ‘total testing process’ in primary care. | Minor |
| Martínez-González et al (2014) Substitution of physicians by nurses in primary care: a systematic review and meta-analysis | To investigate the clinical effectiveness and costs of nurses working as substitutes for physicians in primary care. | Minor |
| Martin-Misener et al (2015) Cost-effectiveness of nurse practitioners in primary and specialised ambulatory care: systematic review | To determine the cost-effectiveness of nurse practitioners delivering primary and specialised ambulatory care. | Minor |
| McNab et al (2017) Systematic review and meta-analysis of the effectiveness of pharmacistled medication reconciliation in the community after hospital discharge | To evaluate the effectiveness of pharmacist-led medication reconciliation in the community after hospital discharge. | Minor |
| Mitchell et al (2001) A descriptive feast but an evaluative famine: systematic review of published articles on primary care computing during 1980-97 | To appraise findings from studies examining the impact of computers on primary care consultations. | Important |
| Moe et al (2017) Effectiveness of Interventions to Decrease Emergency Department Visits by Adult Frequent Users: A Systematic Review. | To evaluate effectiveness of interventions targeting adult frequent ED users. | Important |
| Mold et al (2015) Patients’ online access to their electronic health records and linked online services: a systematic review in primary care | To assess the impact of providing patients with access to their general practice electronic health records (EHR) and other EHR-linked online services. | Important |
| Morgan et al (2013) Non-emergency department interventions to reduce ED utilization: A systematic review. | To assess interventions based outside the ED aimed at reducing ED use. | Important |
| Morley et al (2018) Emergency department crowding: A systematic review of causes, consequences and solutions | To assess literature on the causes and consequences of, and solutions to, emergency department crowding. | Important |
| Nguyen and Sobieraj (2017) The impact of appointment‐based medication synchronization on medication taking behaviour and health outcomes: A systematic review | To assess the impact of the appointment‐based medication synchronisation. | Important |
| Nijmeijer et al (2014) Is franchising in health care valuable? A systematic review | To review of literature on the outcomes of franchising in healthcare. | Minor |
| O’Brien et al (2007) Educational outreach visits: effects on professional practice and health care outcomes | To assess the effects of educational outreach visits (EOVs) on health professional practice and patient outcomes | Important |
| Odendaal et al (2018) Contracting out to improve the use of clinical health services and health outcomes in low‐ and middle‐income countries | To assess effects of contracting out governmental clinical health services to non-governmental service provider/s. | Minor |
| Okumura et al (2014) Assessment of pharmacist-led patient counseling in randomized controlled trials: a systematic review | To review the structure, processes and technical contents of pharmacists’ counselling interventions reported in RCT that had positive health-related outcomes. | Important |
| Pande et al (2013) The effect of pharmacist-provided non-dispensing services on patient outcomes, health service utilisation and costs in low- and middle-income countries. | To examine the effect of pharmacist-provided non-dispensing services in low-and middle-income countries. | Important |
| Park et al (2017) The Effect of Formulary Restrictions on Patient and Payer Outcomes: A Systematic Literature Review | To conduct a systematic literature review that assesses the effects of formulary restrictions on the following outcomes: medication adherence, clinical outcomes, treatment satisfaction, drug utilisation, healthcare resource utilisation, and economic outcomes. | Minor |
| Parmelli et al (2011) The effectiveness of strategies to change organisational culture to improve healthcare performance: a systematic review | To determine the effectiveness of strategies to change organisational culture in improving healthcare performance and to examine the effectiveness of these strategies according to different patterns of organisational culture. | Minor |
| Paudyal et al (2013) Are pharmacy-based minor ailment schemes a substitute for other service providers? A systematic review | To explore the effect of pharmacy-based minor ailment schemes as a substitute for other service providers. | Important |
| Pennington et al (2013) Cost-effectiveness of health-related lifestyle advice delivered by peer or lay advisors: synthesis of evidence from a systematic review | To systematically examine the evidence on the effectiveness of HRLAs and to determine in which areas they are likely to be cost-effective. | Minor |
| Petersen et al (2006) Does Pay-for-Performance Improve the Quality of Health Care? Effectiveness of Pay-for-Performance. | To assess the effect of explicit financial incentives for improved performance on measures of healthcare quality. | Important |
| Pettigrew et al (2018) The impact of new forms of large-scale general practice provider collaborations on England’s NHS: a systematic review | To review the evidence of the impact of new forms of large-scale general practice provider collaborations in England. | Important |
| Randall et al (2017) Impact of community based nurse-led clinics on patient outcomes, patient satisfaction, patient access and cost-effectiveness: A systematic review | To identify the impact of nurse-led clinics. | Important |
| Randell et al (2007) Effects of computerized decision support systems on nursing performance and patient outcomes: a systematic review. | To examine the effects of computerised decision support systems (CDSSs) on nursing performance and patient outcomes. | Important |
| Ranji et al (2008) Interventions to Reduce Unnecessary Antibiotic Prescribing: A Systematic Review and Quantitative Analysis. | To assess the effectiveness of quality improvement (QI) strategies to reduce antibiotic prescribing for acute outpatient illnesses for which antibiotics are often inappropriately prescribed. | Important |
| Rashidian et al (2015) Pharmaceutical policies: effects of financial incentives for prescribers | To determine the effects of pharmaceutical policies using financial incentives to influence prescribers’ practices. | Minor |
| Raven et al (2016) The Effectiveness of Emergency Department Visit Reduction Programs: A Systematic Review | To determine the effectiveness of emergency department visit reduction programmes. | Important |
| Reddy et al (2017) A Systematic Review of the Impact of Healthcare Reforms on Access to Emergency Department and Elective Surgery Services: 1994–2014 | To identify whether healthcare reforms led to improvement in the emergency department (ED) length of stay (LOS) and elective surgery (ES) access in Australia, Canada, New Zealand, and the United Kingdom. | Minor |
| Rose et al (2011) Advanced Access Scheduling Outcomes A Systematic Review | To describe outcomes resulting from implementation of advanced access scheduling in the primary care setting. | Important |
| Rowe et al (2011) The Role of Triage Liaison Physicians on Mitigating Overcrowding in Emergency Departments: A Systematic Review | To examine the effectiveness of triage liaison physicians (TLPs) on mitigating the effects of emergency department (ED) overcrowding. | Important |
| Rowe et al (2011) The Role of Triage Nurse Ordering on Mitigating Overcrowding in Emergency Departments: A Systematic Review | To examine the effectiveness of triage nurse ordering (TNO) on mitigating the effect of emergency department (ED) overcrowding. | Minor |
| Royal et al (2006) Interventions in primary care to reduce medication related adverse events and hospital admissions: systematic review and meta-analysis | To evaluate interventions in primary care aimed at reducing medication-related adverse events. | Important |
| Rudin et al (2014) Usage and effect of health information exchange: a systematic review. | To systematically review and evaluate evidence of the use of health information exchange and its effects on clinical care. | Important |
| Rutebemberwa et al (2014) Financial interventions and movement restrictions for managing the movement of health workers between public and private organizations in low‐ and middle‐income countries | To assess the effects of financial incentives and movement restriction interventions to manage the movement of health workers between public and private organisations in low- and middle-income countries. | Minor |
| Saxon et al (2014) Extended roles for allied health professionals: an updated systematic review of the evidence | To assess the effects of extended roles for allied health professionals. | Minor |
| Soril et al (2015) Reducing Frequent Visits to the Emergency Department: A Systematic Review of Interventions | To establish the effectiveness of interventions to reduce frequent emergency department (ED) use among a general adult high ED-use population. | Minor |
| Souza et al (2011) Computerized clinical decision support systems for primary preventive care: A decision-maker-researcher partnership systematic review of effects on process of care and patient outcomes | To review RCTs assessing the effects of computerised clinical decision support systems for primary preventive care. | Important |
| Studnek et al (2018) Effect of Task Load Interventions on Fatigue in Emergency Medical Services Personnel and Other Shift Workers: A Systematic Review | To assess the effect of task load interventions on fatigue in EMS personnel and other shift workers. | Minor |
| Swan et al (2015) Quality of primary care by advanced practice nurses: a systematic review. | To review RCTs on the safety and effectiveness of primary care provided by advanced practice nurses (APNs) and to evaluate the potential for their deployment to help alleviate primary care shortages. | Important |
| Tan et al (2014) Pharmacist services provided in general practice clinics: A systematic review and meta-analysis. | Review the effectiveness of clinical pharmacist services delivered in primary care general practice clinics | Important |
| Van Herck et al (2010) Systematic review: Effects, design choices, and context of pay-for-performance in health care | To assess the evidence on pay-for-performance in healthcare. | Important |
| Vaughan et al (2015) Costs and cost-effectiveness of community health workers: evidence from a literature review | To review evidence on costs and cost-effectiveness of community health worker (CHW) programmes in low- and middle-income countries (LMICs). | Minor |
| Verma et al (2015) A systematic review of strategies to recruit and retain primary care doctors | To evaluate interventions and strategies used to recruit and retain primary care doctors internationally. | Important |
| Wade et al (2010) A systematic review of economic analyses of telehealth services using real time video communication | To assesses the economic value of synchronous or real time video communication (a type of telehealth delivery). | Important |
| Wallace et al (2015) Impact analysis studies of clinical prediction rules relevant to primary care: A systematic review | To narratively review and critically appraise impact analysis studies of clinical prediction rules relevant to primary care. | Minor |
| Ward et al (2015) Systematic review of telemedicine applications in emergency rooms | To synthesise the existing evidence on the impact of tele- emergency applications that could inform future efforts and research in this area. | Minor |
| Watkins et al (2015) Effectiveness of implementation strategies for clinical guidelines to community pharmacy: a systematic review | To assess implementation strategies for clinical guidelines to community pharmacy. | Minor |
| West et al (2014) A systematic review of the literature on ‘medication wastage’: an exploration of causative factors and effect of interventions | To assess the available evidence on the possible causative factors associated with medication wastage and the effectiveness of any interventions focusing on wastage reduction as an outcome measure. | Important |
| Wilson et al (2016) Interventions to increase or decrease the length of primary care physicians' consultation | To assess the effects of interventions to alter the length of primary care physician consultations. | Minor |
| Wiysonge et al (2016) Public stewardship of private for-profit healthcare providers in low- and middle-income countries | To assess the effects of public sector regulation, training, or co-ordination of the private for-profit health sector in low- and middle-income countries. | Minor |
| Xue et al (2016) Impact of state nurse practitioner scope-of-practice regulation on health care delivery: Systematic review | To examine the impact of state nurse practitioner scope-of-practice regulation on healthcare delivery. | Minor |
| Yuan et al (2017) Payment methods for outpatient care facilities | To assess the effect of different payment systems for outpatient care facilities. | Minor |

# Reviews of reviews

| Publication | Main Focus Area |
| --- | --- |
| Alderdice et al (2013) A systematic review of systematic reviews of interventions to improve maternal mental health and well-being | Service Delivery - Patient support |
| Black et al (2011) The Impact of eHealth on the Quality and Safety of Health Care: A Systematic Overview | Information Technology - Communications and remote health technologies |
| Buja et al (2018) Developing a new clinical governance framework for chronic diseases in primary care: an umbrella review | Governance - Accountability and evaluation |
| Chauhan et al (2017) Behavior change interventions and policies influencing primary healthcare professionals’ practice—an overview of reviews | Workforce - Support |
| Ciapponi et al (2017) Delivery arrangements for health systems in low‐income countries: an overview of systematic reviews | Service Delivery - Patient support |
| Damery et al (2016) Does integrated care reduce hospital activity for patients with chronic diseases? An umbrella review of systematic reviews | Service Delivery - Patient support |
| Eijkenaar et al (2013) Effects of pay for performance in health care: A systematic review of systematic reviews | Financing - Contracting |
| Ekeland et al (2010) Effectiveness of telemedicine: A systematic review of reviews | Information Technology - Communications and remote health technologies |
| Flodgren et al (2011) An overview of reviews evaluating the effectiveness of financial incentives in changing healthcare professional behaviours and patient outcomes | Financing - Contracting |
| Franx et al (2008) Organizational Change to Transfer Knowledge and Improve Quality and Outcomes of Care for Patients with Severe Mental Illness: A Systematic Overview of Reviews | Governance - Accountability and evaluation |
| Hanlon et al (2017) Telehealth Interventions to Support Self-Management of Long-Term Conditions: A Systematic Metareview of Diabetes, Heart Failure, Asthma, Chronic Obstructive Pulmonary Disease, and Cancer | Information Technology - Communications and remote health technologies |
| Herrera et al (2017) Governance arrangements for health systems in low‐income countries: an overview of systematic reviews | Governance - Accountability and evaluation |
| Hillier-Brown et al (2017) The effects of community pharmacy public health interventions on population health and health inequalities: a systematic review of reviews protocol | Workforce - (Pharmacy) Collaboration, Contribution and Substitution |
| Honarvar et al (2019) Opportunities and Threats of Electronic Health in Management of Diabetes Mellitus An Umbrella Review of Systematic Review and Meta-Analysis Studies | Information Technology - Communications and remote health technologies |
| Househ (2014) The role of short messaging service in supporting the delivery of healthcare: An umbrella systematic review | Information Technology - Communications and remote health technologies |
| Joo and Huber (2019) Case Management Effectiveness on Health Care Utilization Outcomes A Systematic Review of Reviews. | Service Delivery - Patient support |
| Lamming et al (2017) What do we know about brief interventions for physical activity that could be delivered in primary care consultations? A systematic review of reviews | Service Delivery - Patient support |
| Lau et al (2015) Achieving change in primary care—effectiveness of strategies for improving implementation of complex interventions: systematic review of reviews | Service Delivery - Patient support |
| Matthys et al (2017) An overview of systematic reviews on the collaboration between physicians and nurses and the impact on patient outcomes: what can we learn in primary care? | Workforce - (Nursing) Collaboration, Contribution and Substitution |
| Mbemba et al (2013) Interventions for supporting nurse retention in rural and remote areas: an umbrella review | Workforce - (Nursing) Collaboration, Contribution and Substitution |
| Mossialos et al (2013) Expanding the role of community pharmacists: Policymaking in the absence of policy-relevant evidence? | Workforce - (Pharmacy) Collaboration, Contribution and Substitution |
| Murphy et al (2017) Clinical-effectiveness of self-management interventions in chronic obstructive pulmonary disease: An overview of reviews. | Service Delivery - Patient support |
| Pantoja et al (2017) Implementation strategies for health systems in low-income countries: an overview of systematic reviews | Service Delivery - Patient support;  Service delivery - Workforce |
| Purcell et al (2014) Telemonitoring can assist in managing cardiovascular disease in primary care: a systematic review of systematic reviews | Information Technology – Surveillance and diagnostic tools |
| San-Juan-Rodriguez et al (2018) Impact of community pharmacist-provided preventive services on clinical, utilization, and economic outcomes: An umbrella review | Workforce - (Pharmacy) Collaboration, Contribution and Substitution |
| Stewart et al (2017) Future perspectives on nonmedical prescribing | Service Delivery - Patient support |
| Wiysonge et al (2017) Financial arrangements for health systems in low‐income countries: an overview of systematic reviews | Coverage - General Financing - General |
| Yeung et al (2016) Integrated multidisciplinary care for the management of chronic conditions in adults: an overview of reviews and an example of using indirect evidence to inform clinical practice recommendations in the field of rare diseases | Service Delivery - Patient support |
