# Supplementary material for: Economic Aspects of Delivering Primary Care Services: An Evidence Synthesis to Inform Policy and Research Priorities
Source: Milbank Q. 2021 Sep 2;99(4):974–1023. doi: 10.1111/1468-0009.12536 (PMC8718591; doi:10.1111/1468-0009.12536)
Supplement: Supplementary file 3 — Findings [file MILQ-99-974-s001.docx]

# Economic Aspects of Delivering Primary Care Services – An Evidence Synthesis to Inform Policy and Research Priorities (Supplementary File 3 - Findings)

This supplementary file provides granular information on the findings of included systematic reviews.^[[1]](#footnote-2)^ We report findings under the following headings:

1. Coverage (x3)
2. Financing (x11)
3. Workforce (x32)
4. Infrastructure (x2)
5. Information & Technology (x13)
6. Patient Supports (x41)
7. Governance (x7)

We complement this information with three summary tables of information sorted according to the structure provided by our taxonomy:

- Table 1 highlights the main focus area of included reviews.
- Table 2 summarises unclear/inconclusive findings highlighted by included reviews.
- Table 3 outlines gaps in evidence, sorted by primary research and systematic reviews.

# Coverage

We identified three reviews which mainly focused on evidence involving arrangements supporting the coverage of primary care services. Available evidence offers uncertain conclusions about the impacts of formulary restrictions and differential user charges for primary and secondary healthcare on costs and healthcare utilisation.

- Authors of a review of eight studies concluded that there was little and very low-quality evidence available that user charges shift use from secondary to primary care and no studies reported on unintended consequences, such as forgone healthcare due to cost.^1^
- In a review of 59 studies, with 42 reporting economic outcomes, authors concluded that there was mixed evidence on formulary restrictions, and suggested that cost savings associated with observed decreases in drug use may be offset by increased healthcare use and its associated costs.^2^
- We identified one review on coverage with important limitations. It assessed 29 studies on reimbursement restriction policies and available evidence suggested that if drugs have cheaper, effective alternatives that target symptoms, such polices can improve cost-effective medication use without negative health effects or increased use of other health services.^3^

# Financing

We identified 11 reviews which mainly focused on evidence involving arrangements to support financing of primary care services. Most focused on the use of different contracting mechanisms – particularly incentive systems – for members of the primary care workforce. Other reviews focused on policies and intervention measures that shifted patients’ costs of accessing primary care support and public procurement of services from private providers. We did not identify any reviews which assessed methods of resource allocation for delivering primary care services.

## Contracting mechanisms.

The evidence reviewed suggested that there are a few contracting mechanisms which may have positive^[[2]](#footnote-3)^ economic impacts, including: drug budgets for pharmacists for modestly reducing drug use, using prospective payment models to increase the clinical activity of primary care dentists, shifting from enhanced fee-for-service models to blended capitation payments to improve provider workloads, and using pay-for-performance incentives and capitated payments to encourage more economic delivery approaches by primary healthcare providers (significant uncertainty).

- A review of 14 studies, including nine reporting on payment models, found high-quality evidence that moving to blended capitation payments from fee-for-service could reduce the number of patients seen per day, with no effect on enrolment or days worked per year.^4^
- In a review of 18 studies, including 12 reporting on costs, authors concluded that the effects of drug budgets can modestly reduce drug use. However, impacts on drug costs and healthcare utilisation were uncertain, and the effects of pay-for-performance and reimbursement rate reduction for pharmacists were also uncertain.^5^
- In a review of two studies looking at the impact of different methods of remuneration on the behaviour of primary care dentists (both of which reported costs), authors noted the potential for supplier induced demand and concluded that further experimental research is required to compare different forms of remuneration and their resource implications.^6^
- In a review of 21 studies, two of which reported costs, evidence suggested that pay-for-performance incentives may improve physician use of treatments and tests, but there is no clear link to improvements in patients’ healthcare utilisation or outcomes.^7^ Authors also concluded, based on low quality evidence, that capitation drove down costs relative to fee-for-service approaches.

The effects of pay-for-performance programmes in ambulatory settings are uncertain, but the intervention may support decreased healthcare utilisation in specific circumstances.

- In a review of 69 studies, with 12 reporting on utilisation, authors identified mixed evidence and noted that several favourable studies lacked rigorous methods. This review emulated earlier systematic reviews on pay-for-performance that concluded that despite some observed positive economic impacts, schemes did not meet the enthusiasm needed for their wholesale adoption.^8,9^

We identified three reviews on contracting that had important limitations.

- Authors of each review found inconclusive evidence and persistent research gaps on the impact of pay-for-performance remuneration schemes.^10–12^

## Procurement mechanisms.

The effects of contracting-out health services, commercial franchising of health services and social franchising of health services are uncertain.

- Contracting-out health services may reduce out-of-pocking spending on curative care, but the implications for adverse effects or for broader societal impacts remain uncertain. Authors of a review which included one study reporting economic outcomes concluded that, when support was necessary, contracting out government-run health services in low- and middle-income countries to private actors made little or no difference to other important health system utilisation or on health outcomes.^13^
- Commercial franchising by private organisations delivering care could offer equal or greater healthcare utilisation and client volumes compared to non-franchised healthcare entities. However, authors of the review, which included seven studies reporting resource outcomes, noted that the two studies which examined care costs per client offered mixed evidence between country settings.^14^

We identified one review with important limitations

- A 2009 review on social franchising — private organisations delivering care with a focus on social benefits — found no evidence that met inclusion criteria.^15^

## Resource Allocations

No reviews met inclusion criteria which addressed resource allocations as part of the financing of primary care services.

# Workforce

We identified 32 reviews that mainly focused on evidence involving workforce-related arrangements of delivering primary care services. Publications examined contributions offered by community health workers, nursing professionals, pharmacists, and physician assistants. Reviews also looked at task-shifting, and policies to address workforce fatigue, recruitment, and training.

## Collaboration, Contribution and Substitution.

Evidence suggests that placing a senior doctor at triage could reduce waiting times for patients by 15-30 minutes compared to a standard single nurse.

- Authors of a review including 22 studies reporting on resource use outcomes concluded cost implications were uncertain, as they were only reported in one study (which reported positive outcomes).^16^ Authors highlighted the need for future studies to evaluate the costs and sustainability of such policies.

Evidence suggests nurse-led activities in ambulatory care settings could reduce waiting times and improve patient flows, but the evidence for overall economic effects are uncertain and available data suggest a mix of positive and negative outcomes.

- Authors of a review, including five studies which reported waiting times, concluded that nurse-initiated medications in the emergency department enabled patients to be seen quicker and are generally safe, but further research on patient cohorts and more robust study designs are needed.^17^
- Authors of a review of 11 studies reporting on resource use concluded that having nurses operate in alternative provider ambulatory primary care roles can save costs and lead to equivalent or better patient outcomes.^18^ Evidence was of mixed quality and cost-effectiveness analyses were limited, but a meta-analysis of two studies found health service costs per consultation were €6.41 lower on average (2006 euros) when nurses operated in ambulatory primary care roles.
- Based on observed consultation times, nurse prescribing may not be an effective substitute for physician prescribing. Authors of a review including eight studies reporting on nurse consultation times concluded that evidence on economic outcomes was lacking and that nurses spent more time on consultations with similar or better outcomes.^19^

Publications focused on expanding nurse practitioner scope of practice regulations can increase the labour supply of nurses, but evidence on costs are mixed; outcomes were uncertain in studies which assessed nurse-led interventions in ambulatory and/or community care, nurse substitution for physicians in primary care and nurse practitioners delivery of hospital-to-community transitional care.

- Authors of a review of 16 studies, which identified the workforce, utilisation, and cost impacts of changes to nurse practitioner scope of practice regulations, concluded that granting nurses greater scope of practice authority was a promising approach to address healthcare demands in rural and medically underserved communities.^20^
- Authors of a review of 16 studies, which reported resource use of nurse-led interventions in ambulatory and/or community care, stated that low-quality economic evaluations and inconclusive findings precluded statements on whether nurse-led interventions had positive economic impacts.^21^
- Authors of a review including nine studies which reported on costs and nine which reported on healthcare utilisation, found inconclusive evidence on whether nurse substitution for physicians working in primary care was associated with substantial savings and/or improved cost-effectiveness.^22^ Such substitution is often referred to “task-shifting”, which involves moving specific tasks, where appropriate, to health workers with less comprehensive training and qualifications.^23^ Savings on salaries may be offset by longer consultations, though better patient outcomes may lead to better medium- to long-term cost reductions.
  - The review had similarities with an earlier publication which was excluded because of its likeness.^24^
- Authors of a review including four studies which reported on healthcare utilisation associated with nurse practitioner delivery of hospital-to-community transitional care found low-quality evidence that displayed either equal or inconclusive outcomes when compared to usual care.^25^

We identified three reviews on nurse-led interventions that had important limitations.

- A review of ten studies concluded that advanced practice nurses appear to offer comparable quality care at equal or lower cost than physicians do. This approach of utilising advanced practice nurses over physicians could potentially alleviate primary care shortages, but further research needs to be done to investigate the effects on additional healthcare use and the impacts on the delivery of appropriate preventive care.^26^
- A review including nine studies reporting on wait times, and one reporting on costs, concluded that emergency nurse practitioner services may positively impact patient waiting times, but the effects on costs are uncertain.^27^
- A review of 15 studies on the impact of nurse-led clinics found evidence indicating that such clinics were more affordable and convenient, and that they reduced overall patient waiting times compared to when only doctor-led clinics were available. Two studies examined cost-effectiveness, and both reported positive outcomes. However, there was limited reporting on whether nurse-led clinics were cost-equivalent to, or more cost-effective than, standard physician-led clinics. Further standardised research should assess the economic value of nurse-led clinics.^28^

We identified five reviews on pharmacist-led non-dispensing services that all had important limitations.

- Authors of a review of 14 studies concluded that pharmacist-led non-dispensing services appear to lead to little or no difference in hospital attendance or admissions, with little perceived difference in the effectiveness of care. This finding has important implications for future substitution practices. ^29^
- A review of 31 studies on pharmacist-led non-dispensing services suggested that this type of care is appropriate, and that consultations are less expensive than those with primary care physicians. However, it remained unclear clear how much the schemes shifted demand for these interventions away from general practice settings.^30^
- A review including two studies reporting on costs found that co-locating clinical pharmacist services in primary care general practice clinics tended to have limited or no effects on medical costs.^31^
- A review of pharmacist-led counselling and education interventions, which included four studies reporting economic outcomes, did not clearly synthesise study results and had inconclusive findings. ^32^
- Authors of a review on non-dispensing pharmacist services included four studies reporting healthcare utilisation and one study reporting costs from lower- and upper-middle income countries. Authors concluded that while the evidence is uncertain, it indicates that these non-dispensing activities may lead to cost-reductions and make the case for further research.^33^

We identified two reviews on the roles of physician assistants in primary care; both had important limitations.

- A review including four studies reporting economic outcomes found that existing research allows few conclusions on whether physician assistants in emergency rooms are cost-effective or improve patient flows. However, indirect evidence suggests cost implications warranting further research.^34^
- A review including 18 studies reporting financial, workflow and retention outcomes, showed available evidence provides little in the way of comparative or economic analysis to inform recommendations for physician assistants in primary care settings who support general practitioners and family physicians. Authors noted that there is an apparent trade-off between supervision and task-shifting involved with physician assistants’ presence in the primary care setting.^35^

We identified one review focused on health-related lifestyle advice delivered by peer or lay advisors.

- Evidence suggested that there is limited evidence on the resource impacts of hiring peer or lay advisors. What evidence is available suggests that these advisors are only cost-effective if they target patient behaviours which are likely to have a large impact on overall health-related quality of life.^36^

We identified reviews describing positive economic impacts associated with community health workers and uncertain outcomes for allied health professionals and dental auxiliaries.

- Community health worker programmes in low- and middle-income countries appear to be cost-effective from health systems and societal cost perspectives. A review of 32 studies reporting costs and cost-effectiveness concluded that such programmes can be cost-effective in certain settings. The authors also highlighted that more attention needs to be devoted, when developing or scaling up programmes, to assessing economic outcomes from government and societal perspectives, and to determining why such programmes are cost-effective in some settings, but not in others.^37^
- We identified one review with important limitations on the roles of allied health professionals, such as physiotherapy, occupational therapy, and speech pathology in primary care spaces. A review including three studies reporting cost-effectiveness concluded that extended roles for allied health professionals may be cost-effective, but a limited evidence-base hinders recommendations.^38^
- The economic impacts of dental auxiliaries substituting for dentists are uncertain. A review of available evidence did not identify any studies which reported on economic outcomes and the only evidence that was available was outdated and limited in scale, content, quality and generalisability.^39^

## Demand, Supply, and Training

Publications which focused on workforce fatigue management offered uncertain conclusions about task load interventions and biomathematical models for fatigue mitigation.

- Authors of a review of five studies reporting on task load interventions for emergency medical service personnel and other shift workers did not identify evidence on costs.^40^ They also highlighted considerable gaps in the scientific literature about operational decisions on changing task loads for emergency medical service personnel.
- Authors of a review which included one study on using biomathematical models for emergency medical service personnel to support fatigue mitigation found uncertain, but favourable, evidence on system-level cost impacts. Reviewers highlighted the need for more research in an area that has seen widespread commercial interest and investment.^41^

Publications focused on the recruitment and retainment of primary care physicians found varied outcomes.

- Well-organised and well-funded rural clinical school/placement programmes can improve rural graduate employment of medical students in primary care.^42^ Authors of a review of 62 studies examining the effectiveness of rural placement and rural education programs within medical education concluded that they yield increased rates of intention towards and actual graduate rural employment.^42^ For long-term retention, two studies reported positive rates, though five lower-quality studies showed mixed results.

One review suggested that rural recruitment campaigns may improve recruitment and retainment of primary care doctors, but the review had important limitations and the evidence included was low-quality.

- Authors of a review of 51 studies on various recruitment approaches noted that while there was weak and mixed evidence on the effects of financial incentives, success rates may improve when incentives are tied to working in rural areas, long service obligations, and flexibility in career options.^43^ Authors also concluded that video marketing may have a negative effect on recruitment and retainment of physicians. Insufficient quality evidence was available to assess the utility and impacts of returner schemes, retainer schemes, re-entry schemes, international recruitment, specialised recruitment, support for professional development or research, delayed partnerships, wellbeing and peer support or mixed approaches.

We identified one review on training professionals involved in primary care delivery that had important limitations.

- A review of seven studies which reported economic outcomes of these training interventions suggested that savings could exceed costs, but authors noted it was unclear if performance might deteriorate over time and whether repeated visits are good value for money.^44^

We identified one review on the implications of the feminisation of the primary care physician workforce on service supply.

- Available evidence indicates that once the effect of family characteristics has been accounted for, physician sex has no effect on time spent working. Further research is warranted on work-life balance, caregiving and childrearing responsibilities.^45^

We identified one review on financial interventions and restrictions on the movement of health workers between public and private organisations in low‐ and middle‐income countries.

- It did not identify any eligible studies.^46^

## Staff Support Activities

Publications focused on measures to support staff working in the delivery of primary care services included several interventions.

- Tailored strategies for improving professional practice and health outcomes can be effective in improving the processes of care; however, the effects are variable and tend to be small to moderate. Effects of other measures are uncertain. Authors of a review of 32 studies on tailored strategies highlighted that heterogeneous interventions and measurements, combined with limited research, reduce clarity around what the effects of an optimally tailored intervention are.^47^
- The economic impacts of interventions aimed at changing organisational culture are unclear. Authors of a review including one study reporting effects related to staff supply and retention concluded that available evidence on improving healthcare performance was limited and of low quality.^48^
- The economic impacts of management interventions for dual practice by health workers – that is where health workers hold two or more jobs – is unclear. Authors of a review of dual practice management interventions did not identify any studies eligible for inclusion.^49^

# Infrastructure

We did not identify systematic reviews or meta-analyses which mainly focused on evidence involving interventions or policies related to infrastructure, including physical access, fixed capital assets, or resilience measures. We identified one review which examined the effects of scaling up primary care facilities. This review had uncertain findings.

- A review with important limitations, including four studies in England which reported on process outcomes, found that scaling up may result in improved care quality, but may also increase staff turnover and requires substantial financial investments.^50^

We identified one review focused on surveillance and diagnostic tools, which found that these can support the workload of physicians conducting laboratory testing in primary care, but effects on costs remain uncertain. ^51^

- Authors of a review of 22 studies reporting on outcomes including workload, turnaround times, and costs concluded that IT solutions could improve workload related elements, but the review did not synthesise cost conclusions of included studies.^51^

# Information & Technology

We identified 13 reviews which mainly focused on evidence involving the involvement of information & technology in the delivery of primary care services. Publications focused on communication and remote health technologies, health records and decision support tools.

## Communications and remote health technologies

Publications focused on mHealth and telemedicine interventions offered positive conclusions about their economic impacts, though impacts of interactive telemedicine and telephone consultations are less certain.

- Authors of a review of 39 studies using a variety of costing perspectives concluded that most identified evidence supported the cost-effectiveness of mHealth interventions across many uses. However, they cautioned that applications of mHealth be considered on a case-by-case basis and noted that research set in low- and middle-income countries is lacking – representing less than 15% of included studies.^52^
- These findings are complemented by a review of 17 economic evaluations of telemedicine in Japan that found most studies indicated positive levels of economic efficiency and highlighted the need for better adoption of economic evaluation standards, rigorous empirical evidence and assessments of willingness to pay for interventions.^53^

The effect of telehealth on secondary healthcare use is uncertain, with a minority of the evidence suggesting significant decreases.

- Authors of a review of 22 studies concluded that disparities in study findings were likely due to the variability of examined telehealth programmes and contextual factors influencing programme effectiveness.^54^
- The effects of interactive telemedicine are also uncertain. Authors of a review of studies, 54 of which reported on resource use and 26 of which reported on costs, concluded that evidence was mixed for healthcare resource use, with changes in hospital admissions ranging from a relative decrease of 64% to an increase of 60%. Different approaches to measuring costs precluded synthesis of economic outcomes reported by studies.^55^
- The effects of telephone consultations in general practice are uncertain, but they may reduce general practice facility workloads. Authors of a review including evidence from a single study and two systematic reviews concluded that establishing clear resource impacts required further research.^56^

We identified two reviews on using remote technologies to delivery primary care services that both had important limitations.

- A review including three studies reporting economic outcomes of teledentistry found that teledentistry could be cost saving when compared with conventional dentistry. Authors noted that there has been a persistent absence of good-quality economic studies in this field of research.^57^
- A review of 36 economic analyses on real time video communication suggested that, from the health system, provider and patient perspective, using the approach for health service delivery was cost-effective for home care and provided access to on-call hospital specialists, had mixed results for rural service delivery, and was not cost-effective for local delivery of services between hospitals and primary care.^58^

## Health records and decision supports

We identified three reviews focused on healthcare decision supports, two had important limitations.

- The effects of clinical prediction rules relevant to primary care are uncertain, but evidence suggests that these may reduce inappropriate admissions and prescribing. Authors of a review of 18 studies reported that limited research has been conducted on the impact of clinical tools that aim to simplify and standardise clinician decisions.^59^
- A review including six studies which reported the cost impacts of computerised decision support systems found positive, but uncertain evidence. While systems may reduce some costs of care, authors suggested that that it was not possible to draw definitive conclusions based on the evidence from available studies.^60^
- A review of eight studies found that computerised decision support systems for nurses could support workload reductions, but given the presence of “enormous unexplained variation between health professionals” using the systems, future studies should acknowledge and explore the complexity of their impact.^61^

We identified three reviews focused on health records that had important limitations.

- A review including eight studies reporting on resource use found evidence that suggested a low-strength association between health information exchange and reductions in emergency department costs/utilisation.^62^
- A review of 17 studies reporting on patients’ online access to electronic health records found evidence that suggested these measures can have positive impacts on reducing physician workloads, but that patients had a low willingness to pay for access to online services ^63^.
- A 2001 review including six studies reporting on consultation length found that the use of computers in primary care consultations has uncertain impacts. The review found that while computers could be cost prohibitive and increased or didn’t significantly impact consultation length, these systems could also increase productivity.^64^ No costedv studies were identified.

# Patient Supports

We identified 42 reviews that which mainly focused on evidence involving interventions targeted to improve the delivery of primary care and minimise resource-intensive activities. These included reviews of measures implemented to support medical staff, measures to address unplanned and frequent healthcare usage, and supportive measures to engage patients via general checks, integrated care, medication management and specialist medical care.

## Addressing unplanned and frequent healthcare usage

The evidence on ex-ante measures to reduce unplanned healthcare usage suggests that interdisciplinary team-based models of care and walk-in centres as well as general practice co-operatives can offer supportive care streams.

- Authors of a review of 14 studies, including five reporting on care models, found moderate-quality evidence that using interdisciplinary team-based models of care can reduce emergency department use, with mixed evidence for hospital admissions.^4^
- Authors of a review of 11 studies found limited evidence on the observed efficacy of walk-in centres and general practice co-operatives as alternative care pathways for emergency department attendees. Review authors concluded that further evidence is needed to be confident enough to draw conclusions.^65^

We identified five reviews focused on ex-ante measures to reduce unplanned healthcare usage that had important limitations.

- A review including 13 studies on emergency department visit reduction programmes, with five studies reporting cost data, found that case management of high-risk patients could lower resource impacts, but studies lacked cost-effectiveness information. They also found that co-payments could create significant emergency department visit reductions for low acuity visits. Reviewers highlighted that many emergency department visit reduction arrangements assume that primary care services offer a lower cost care substitute, but noted that evidence suggests emergency departments serve as a safety net for overburdened primary care systems.^66^
- A review including 39 studies on reducing emergency department utilisation included eight studies which reported cost data.^67^ Authors concluded most patient financial incentives and managed care interventions displayed reductions in emergency department use, with some uncertainty due to evidence quality. Interventions which increase non-emergency department capacity could also reduce use – though in one study, evidence suggested uncertain outcomes as increased capacity led to supplier-induced over-demand. Poor-quality studies throughout the evidence base inhibited general conclusions.
- A review of 48 studies assessed organisational interventions targeting reduced emergency department utilisation.^68^ Authors concluded there was consistent evidence supporting the use of cost-sharing, extra-hospital emergency services and improving primary care accessibility to reduce emergency department utilisation. Filter interventions, such as primary care gatekeeping were found to have little impact on emergency department visits. Educational interventions were found to have positive impacts on reductions, but for hospitalisation rates rather than emergency department rates.
- A review including 44 studies on factors and interventions in primary care linked to emergency healthcare utilisation, including four which reported cost data, found that access to primary care reduced emergency healthcare use in the United States and Canada, but effects in European heath systems were uncertain.^69^
- A review of 33 studies reporting on unplanned healthcare use in rural populations found that disease management, telemedicine and community health clinics may lead to reductions in use, but uncertainty remains due to weak methodological rigour across studies. Cost savings were identified in two studies, but were not specified.^70^

Other reviews

- A 2013 review focused on triage systems for pre-hospital emergency medical services identified no eligible studies.^71^
- A review of diversion strategies found no conclusive evidence regarding the impact on emergency department utilisation and subsequent healthcare utilisation. The overall quality of the research limited the ability of this review to draw definitive conclusions and more research is required prior to widespread implementation.^72^
- A review including 34 studies reporting on primary care service interventions targeting reductions in inappropriate emergency department attendance identified no conclusive evidence.^73^ Authors noted the need to assess patient behaviour and whether actual, or perceived, absence of primary care results in increased emergency attendances. Review findings supported the idea that increasing access points for urgent care may unmask latent demand which is more likely to be inappropriate for emergency departments.
- A review including 52 studies reporting on measures to address emergency department crowding identified a variety of interventions reporting positive outcomes, such as whole-of-system initiatives to meet patient disposition time targets and extended hours of primary care services. ^74^ Some studies reported costs, but none reported cost-benefit analysis. Authors noted that due to context specific factors, a ubiquitous approach to the issue was unlikely to be feasible.

The evidence on ex-post measures to reduce unplanned healthcare usage suggests that triage nurse ordering, short-stay units and rapid assessments units can have positive economic impacts, while evidence suggests unclear impacts for locating primary care professionals in hospital emergency departments.

- Authors of a review of 14 studies concluded that triage nurse ordering could reduce emergency department length of stay by over 30-minutes and that such interventions would benefit from the implementation of standardised guidelines.^75^
- Authors of a review of four studies reporting economic outcomes concluded emergency department short-stay units could be cost-saving, but evidence should be interpreted with caution. They also highlighted that cost savings should be integrated into full economic evaluations using more robust statistical and econometric methods.^76^
- Authors of a review including two studies reporting on length of stay concluded that rapid assessment units could reduce overcrowding in emergency departments and improve resource use. However, available studies should be interpreted with caution, as robust conclusions were hindered by the number of participants and methodological quality.^77^
- Authors of a review of four studies, including two reporting cost-effectiveness, found that inconsistent and very low-certainty evidence prevented conclusions about the economic impacts of co-location.^78^

We identified one review focused on ex-post measures to reduce unplanned healthcare usage that had important limitations.

- A review including 19 studies examining resource impacts related to triage liaison physicians found very little quality evidence that the intervention positively impacts resource use.^79^

The evidence on measures to address frequent healthcare usage suggests that case management, individualised care plans and information sharing could lead to modest cost savings when targeted at adult frequent emergency department users. But there appear to be caveats.

- Authors of a review of 17 studies on a range of interventions, including nine reporting costs, concluded that while observed impacts were positive, they were modest among the high-use population and none of the studied interventions appear likely to yield substantial overall cost savings for the healthcare system.^80^

We identified two reviews focused on measures to address frequent healthcare usage that had important limitations.

- A review of 31 studies of interventions targeted at adult frequent emergency department users noted that interventions can effectively decrease healthcare utilisation and costs. However, authors did not offer a summary of which specific interventions, such as case management, led to reductions.^81^
  - Previous reviews on the subject highlighted the potential value of case management, individualised care plans, social worker support and use of care coordination plans in decreasing emergency department use.^82,83^
- A review including 17 studies which examined approaches to support frequent attendees in primary care found mixed evidence on primary care utilisation and some positive evidence on hospital utilisation. Authors noted that addressing frequent attendance “does not seem to allow” for simple or uniform procedural approaches and only identified one study reporting costs, which found a disease management programme increased healthcare use, and costs increased in tandem with better health outcomes.^84^

## Patient Support Activities

The evidence on general patient support activities suggested that advanced access scheduling can have positive economic impacts, while public healthcare and private supplier initiatives to systematically offer general health checks to the general population appear to make little or no difference to hospitalisation rates.

- Authors of a review of 24 studies, with important limitations, identified several positive improvements associated with advanced access scheduling in the primary care setting, including reduced waiting times for appointments, lower no-show rates and improved patient continuity. Further research was recommended to assess other impacts including utilisation and loss to follow up.^85^
- Authors of a review, including five studies which reported on healthcare use, concluded that general health checks offered systematically to the general population make little or no difference to hospitalisation rates, are unlikely to prove beneficial and may lead to unnecessary tests and treatments.^86^
- The effects of interventions to alter the length of primary care physicians’ consultations are unclear. Authors of a review including three studies reporting on consultation length could not support or refute their use, due to the short-term nature of changes in consultation time for patients and lack of information which would enable cost assessments.^87^
- Physician consultation time appears to be significantly linked with healthcare spending per capita, primary care physician density and secondary healthcare utilisation for ambulatory-sensitive conditions.^88^

We identified one review focused on general patient support activities that had important limitations.

- A review including three studies which compared healthcare use when telephone consultations for triage were used as an assessment by a health professional. Authors found that the intervention reduced GP surgery contacts and GP out-of-hours visits. However, further evaluation was recommended to measure effects on repeat visits and resource use.^89^

The evidence on integrated care measures suggested that accountable care organisations in the United States can reduce healthcare usage, but the impacts of service integration between healthcare providers appear unclear in other settings.

- Authors of a review of 42 studies on accountable care organisation formation in the United States found evidence suggesting that integrated systems of care can lead to a reduction in emergency and hospital healthcare use. They also noted that there were no studies identified in their review which indicated that cost reduction incentives negatively impacted processes or outcomes of care.^90^
- A review of nine studies on strategies to integrate primary health care services in low- and middle-income settings drew upon studies from India, Nepal, South Africa, Tanzania, Togo, Zambia, and Zimbabwe. Based on the available evidence, authors were unable to conclude that integration had positive economic impacts.^91^
- Authors of a review of 124 studies on integration or coordination programmes between healthcare providers in a range of high-income settings found inconsistent or limited evidence for outcomes including system-wide impacts on primary care, secondary care, and healthcare costs. Authors noted that their review added to the increasing evidence-base that integrated care rarely leads to unequivocally positive effects and may offer contradictory financial incentives to stakeholders.^92^

Publications focused on measures to engage patients in specialist care through primary care services found positive, but uncertain, evidence on the co-location of speciality care services in primary practice settings and unclear findings on whether referring patients to non-clinical support measures (social prescribing) is effective or good value for money.

- Authors of a review including five studies reporting on utilisation and three reporting on costs associated with co-location of speciality care services in primary practice settings concluded that, despite evidence of reduced waiting times and costs of care, further research was warranted due to limited study quality. In addition, it remains unclear whether co-location can significantly affect hospital admission rates.^93^
- Authors of a review including five studies reporting healthcare use and two reporting costs associated with social prescribing were unable to make conclusions about economic impacts. They called for further research to address an evidence gap which has developed despite wide advocation and implementation of social prescribing in the United Kingdom and elsewhere.^94^

We identified two reviews focused on measures to engage patients in specialist care through primary care services that had important limitations.

- A review of nine studies reporting on the impacts of specialist outreach clinics in primary care and rural hospitals found that outreach services can improve efficiency by reducing patient costs and can achieve greater cost-effectiveness through multifaceted interventions. However, authors noted that evidence was “especially deficient” for rural and disadvantaged communities who stood to benefit the most from outreach interventions.^95^
- A review including two economic evaluations on improving outpatient referrals from primary to secondary care found limited evidence that suggested educational interventions may improve referral processes and associated resource use. The effects of organisational and financial interventions are uncertain due to evidence gaps.^96^ These conclusions were “broadly similar” to those of an earlier review.^97^

The evidence on measures to support management of medicine usage suggested the following: fee-for-service pharmacist-led medication reviews can significantly reduce hospitalisation rates, but their impact on healthcare costs is uncertain; information leaflets may reduce antibiotic prescription, use and patients’ intention to re-consult in primary care settings; and pharmacist-led medication reconciliation after hospital discharge appears to be ineffective for reducing workloads of healthcare teams.

- Authors of a narrative review and a meta-analysis, which included seven studies reporting on healthcare utilisation associated with medication reconciliation, concluded available evidence did not support the hypothesis that pharmacists who identify and resolve discrepancies can reduce healthcare team workloads.^98^ Available evidence contained limited measurement of healthcare use and workloads.
- Authors of a review, which included nine studies reporting hospitalisation rates and nine reporting costs, identified positive findings for fee-for-service pharmacist-led medication reviews on healthcare use through meta-analysis. However, authors also found mixed evidence on medical costs, medication costs and costs for health services.^99^
- Authors of a review of eight studies reporting on the information leaflets in primary care settings encouraged their use during consultations for common infections, but noted that their impact on actual re-consultation rates remains unclear.^100^

We identified five reviews focused on measures to support management of medicine usage that had important limitations.

- A review including two studies reporting on resource use and costs found that medication synchronisation appears to carry a positive benefit-cost ratio when it is associated with the treatment of certain chronic conditions. However, there is no clear evidence on the effects of medication synchronisation on hospitalisations.^101^
- A review including two studies reporting on costs and medication wastage made tentative conclusions that instalment dispensing may be a useful approach to reduce the overall general practice drugs bill, but highlighted that evidence was limited.^102^
- A review including 25 studies which reported on the impacts of interventions in primary care to reduce medication-related adverse events identified limited evidence. Authors concluded that, while some studies reported reduced hospital admissions for interventions incorporating medication review, the effect became insignificant when evidence from randomised controlled trials was pooled.^103^
- A review including three studies reporting on costs of reminder and feedback interventions for medication adherence had inconclusive findings on resource outcomes.^104^
- A review including two studies reporting on costs concluded that quality improvement strategies to reduce antibiotic prescribing for acute outpatient illnesses have unclear outcomes. Despite finding evidence of reductions in prescribing costs, a lack of associated cost-benefit analysis, due to interventions not being costed, prevented further conclusions.^105^

# Governance

We identified seven reviews which mainly focused on evidence involving governance strategies for supporting the delivery of primary care services. These included assessments of arrangements involving clinical guidelines, accountability, evaluation and patient engagement.

Included publications suggested that positive impacts, with caveats, of time-based targets and redesigning work environments and timescales in emergency departments, and of measures to implement clinical guidelines for community pharmacies.

- Authors of a review including 13 studies examining the impact of healthcare reforms on emergency department access concluded that redesigning work environments and timescales can have positive outcomes. Well-designed target-setting may also be useful in achieving health reform goals. However, steps should be taken to ensure targets are accompanied by changes to the system to address increased pressure on clinicians that might incentivise manipulation of performance data.^106^
- Authors of a review that identified three studies reporting on economic impacts of implementing clinical guidelines for community pharmacies did not offer synthesised conclusions due to the complexity of implementation science, the heterogeneity of studies and the poor methodological quality of research in the setting. Nonetheless, some evidence suggests that less complicated interventions could be more cost-effective, easier to sustain, and better able to inform future practice. ^107^
- We identified one review focused on antimicrobial resistance stewardship in emergency departments that had important limitations. The review included two studies reporting on resource use and concluded that the stewardship interventions could reduce resource use, but also suggested that available evidence suffered from small sample sizes and poor methodological quality.^108^

Evidence is uncertain on the impact of managerial supervision arrangements including routine supervision of primary healthcare providers and public sector regulation, training, or co-ordination of the private for-profit health sector in low- and middle-income countries.

- Authors of a review on managerial supervision involving routine supervision of primary healthcare providers based their conclusions on nine studies conducted in Benin, Ethiopia, Kenya, South Africa, Zimbabwe, Nepal, Philippines, Thailand, and Brazil. They concluded that there was insufficient high-quality evidence to advocate for any form of supervision based on costs and service utilisation, despite observed performance improvements.^109^ Authors noted that one low-quality study reported that decreasing supervision frequency saved costs without negatively impacting provider performance. Further research needs to be guided by a framework that allows for identification of key supervision components, positive outcomes, and implementation resources.
- It is unclear what the impact of public sector regulation, training, or co-ordination of the private for-profit health sector is in low- and middle-income countries. A review of public sector engagement practices with the private for-profit health sector included three studies set in Vietnam and Kenya which included information on costs. Authors reported that evaluations were not rigorous enough to make economic conclusions and that despite widespread availability of services, there is a shortage of evidence syntheses and available literature on governmental collaboration and regulatory activities with private for-profit providers.^110^

Publications on stakeholder engagement in the delivery of primary care services offered uncertain conclusions about the effect of shared decision-making in emergency departments and patient-mediated interventions. The effect of shared decision-making in emergency departments is unclear, but computerised decision support interventions may reduce healthcare use without risking clinical harm.

- Authors of a review including two studies measuring healthcare use noted that no empirical evidence suggests that shared decision-making cannot be a successful model of care in emergency departments. However, further efforts are required to develop and evaluate the feasibility and acceptability of interventions.^111^
- Authors of a review on patient-mediated interventions, which included one study on the cost-effectiveness of clinical practice adherence, could not make conclusions about resource use because of the lack of robust or comparable evidence.^112^

# Summary Tables

### Table 1 - Focus of included reviews

| Components | Minor limitations | | Important limitations |
| --- | --- | --- | --- |
| Coverage | User fees^1^; formulary restrictions^113^. | | Drug reimbursement^3^. |
| Financing | Payment models and financial incentives^4,5,7–9,114^; contracting out health services^13^; franchising health services^14^. | | Payment models and financial incentives^10–12^;  franchising health services^15^. |
| Service Delivery: Workforce | Task shifting and contributions specifically involving - senior doctors^16^, nurses^17–20,22,24,25^, peer advisors^36^, community health workers ^37^, allied health professionals^38^, dental auxiliaries^39^; recruitment and retainment of primary care physicians^42^; feminisation of the primary care workforce^45^;  management of health worker movement between public and private organisations^46^; management of worker fatigue^40,41^. | | Task shifting specifically involving - nurses^26–28^, pharmacists^29–33^, physicians assistants^34,35^;  training primary care professionals^44^; recruitment and retainment of primary care physicians^43^. |
| Service Delivery: Infrastructure | Lab testing in primary care^51^. | | Size of primary care facilities^50^. |
| Service Delivery: Information Technology | Communications and mobile health technologies^52–54,56,115,116^; decision support tools^59^. | | Communications and mobile health technologies^57,58^; decision support tools^60,61,64^; health records^62,63^. |
| Service Delivery: Patient Supports | Addressing unplanned healthcare use and overcrowding in emergency departments^65,68,71,72,75–78,80^; integrated care^90–92^; medication and prescribing support^98,99^;  patient education^100^; patient reminders^104^; primary care consultations^86–88^; referrals and specialised care^93,94^ improving professional practice^47^; managing dual practice among health workers^49^; managing organisational culture^48^. | Addressing unplanned healthcare use and overcrowding in emergency departments^66,67,69,70,73,74,79,81^; addressing frequent healthcare use^84^; medication and prescribing support^101–103,105^; primary care consultations^89^; primary care scheduling^85^; referrals and specialised care^95,96^. | |
| Governance | Target setting and organisational reforms^106^; clinical guideline implementation^107^; stewardship and supervision^109,117^; shared decision-making^111^; patient‐mediated interventions^112^. | | Antimicrobial stewardship^108^. |

***Important limitations:*** *the results of the review need to interpreted with caution due to limitations, another relevant review should be identified if possible.*

***Note****: Reviews with similar focus areas differed based on the specific arrangements, settings, or outcomes they evaluated.*

### Table 2 – Summary of Unclear/Inconclusive Findings

| Components | Effects Unclear |
| --- | --- |
| Coverage | - Formulary restrictions and differential user charges for primary and secondary healthcare have unclear impacts on costs and healthcare utilisation, including healthcare forgone due to cost.^1^ |
| Financing - Contracting | - Effects of drug budgets on drug costs and healthcare utilisation.^5^ - Pay-for-performance and reimbursement rate reduction for pharmacists,^5^ impacts of different forms of remuneration for dentists,^6^ pay-for-performance incentives for physicians and improvements in patients’ healthcare utilisation or outcomes,^7^ and pay-for-performance programmes in ambulatory settings (some positive economic impacts, but schemes did not meet the enthusiasm needed for their wholesale adoption).^8,9^ - Pay-for-performance remuneration schemes are characterised by inconclusive evidence and persistent research gaps.^10–12^ (IL) |
| Financing - Procurement mechanisms. | - The effects of contracting-out health services,^13^ commercial franchising of health services,^14^ and social franchising of health services are uncertain (IL).^15^ - No reviews met inclusion criteria which addressed resource allocations for care provision as part of the financing of primary care services. |
| Workforce - Collaboration, Contribution and Substitution. | - Cost implications of placing a senior doctor at triage, compared to a standard single nurse.^16^ - Overall economic effects of nurse-led activities in ambulatory care settings, including nurse-initiated medications in the emergency department,^17^ nurse prescribing as a substitute for physician prescribing,^19^ and nurse-led interventions in ambulatory and/or community care,^21^ nurse substitution for physicians working in primary care,^22^ and nurse practitioner delivery of hospital-to-community transitional care when compared to usual care.^25^ - Impacts of advanced practice nurses on healthcare use/delivery of appropriate preventive care,^26^ emergency nurse practitioner services on costs are uncertain,^27^ and the economic value of nurse-led clinics compared to standard physician-led clinics.^28^ (IL) - Economic impacts of physician assistants in emergency rooms^34^ or primary care settings who support general practitioners and family physicians.^35^ (IL) - Resource impacts of overall efforts of hiring peer or lay advisors.^36^ - Economic impacts of dental auxiliaries substituting for dentists.^39^ |
| Workforce - Demand, Supply, and Training | - Publications which focused on workforce fatigue management offered uncertain conclusions about task load interventions for emergency medical service personnel.^40^ - Utility and impacts of rural recruitment activities including returner schemes, retainer schemes, re-entry schemes, international recruitment, specialised recruitment, support for professional development or research, delayed partnerships, wellbeing and peer support or mixed approaches.^43^ (IL) - Financial interventions and restrictions on the movement of health workers between public and private organisations in low‐ and middle‐income countries.^46^ |
| Infrastructure | - Cost impacts of investing in surveillance and diagnostic IT solutions for primary care settings.^51^ - Scaling up the size of primary care facilities.^50^ |
| Information & Technology | - Impacts of mHealth on secondary care use.^54^ - Impacts of interactive telemedicine.^55^ - Comprehensive understanding of the impacts of applying mHealth in different settings, particularly with respect to applications of mHealth across low- and middle-income countries, where research is relatively slim.^52^ |
| Service Delivery | - Impact of interdisciplinary team-based models of care on hospital admissions.^4^ - Efficacy of walk-in centres and general practice co-operatives as alternative care pathways for emergency department attendees.^65^ - Cost-effectiveness of emergency department visit reduction programmes, though effects on resources impacts indicate positive economic impacts.^66^ - Impacts of access to primary care on emergency healthcare use in the United States and Canada in European heath systems.^69^ - Triage systems for pre-hospital emergency medical services identified no eligible studies.^71^ - Impact of diversion strategies on emergency department utilisation and subsequent healthcare utilisation.^72^ - Interventions targeting reductions in inappropriate emergency department attendance.^73^ - Locating primary care professionals in hospital emergency departments or the use of triage liaison physicians.^7879^ - Implementation of uniform approaches to support frequent attendees in primary care.^84^ - Integration of primary care services, with other care services, in settings outside of the United States/Accountable Care Organisation models.^9192^ - Impact of co-location of speciality care services on hospital admission rates.^93^ - Referring patients to non-clinical support measures (social prescribing).^94^ - Organisational and financial interventions to improve outpatient referrals from primary to secondary care.^96^ - Impacts of medication reconciliation and fee-for-service pharmacist-led medication reviews. ^9899^ - Interventions in primary care to reduce medication-related adverse events.^103^ - Quality improvement strategies to reduce antibiotic prescribing for acute outpatient illnesses have unclear outcomes.^105^ |
| Service Delivery – Staff Support Activities | - Tailored strategies for improving professional practice and health outcomes.^47^ - Interventions aimed at changing organisational culture are unclear.^48^ - Management interventions for dual practice by health workers – where health workers hold two or more jobs.^49^ |
| Governance | - Implementation clinical guidelines for community pharmacies (due to the complexity of implementation science, the heterogeneity of studies and the poor methodological quality of research in the setting).^107^ - Impact of managerial supervision arrangements including routine supervision of primary healthcare providers and public sector regulation, training, or co-ordination of the private for-profit health sector in low- and middle-income countries.^109110^ - Stakeholder engagement in the delivery of primary care services, via shared decision-making in emergency departments or patient-mediated interventions.^111112^ |

### Table 3 – Summary of Gaps in Primary Research and Systematic Reviews

| Component | Systematic reviews needed | Primary research needed |
| --- | --- | --- |
| Coverage | • Policies that ensure primary care services are provided for the people that need them.  • Policies that ensure people can access a breadth of primary care services. | • Policies involving formulary restrictions, including in situations where interchangeable drugs are not available.^2^ • Interventions for shifting healthcare use from secondary to primary care providers, including user charges.^1^ |
| Financing | •Approaches to allocating finite resources for primary care services and facilities.  • Collection of funds (community loan schemes, health savings accounts), insurance schemes, purchasing of health services and paying health workers, and financial incentives for the providers and recipients of care.^117^  • Analysis of unintended consequences and variable designs of programs involving financial incentives.^118,119^ | • Arrangements involving financial incentives, such as pay for performance schemes, for practitioners working in primary care settings including general practice, pharmacy, and ambulatory care.^5,7,114^  • Reimbursement rate reduction policies for pharmacists.^5^  • Assessments of the spillover effects of contracting-out health services, including equity implications.  • Private care organisations providing commercial franchising and social franchising.^14,15^ |
| Service Delivery: Workforce | • Professional role expansion or task shifting in general practice and by allied health professionals, movement of health workers between public and private care, and working conditions for health workers. ^120^  • Implementation of workforce policies alongside tailored incentive mechanisms.^121^  • Effective approaches to training, supervising and providing appropriate accountability and ownership structures for community health workers.^122^ | Evaluations of task shifting in primary care, including:  • The implications of nurses: leading care delivery in ambulatory and community care settings, substituting for physicians, expanding their scope of practice, and nurse practitioners delivering hospital to community transitional care.^17,18,21,25^  • Counselling and education led by pharmacists.^32^  • Services provided by allied health professionals, dental auxiliaries, and physician assistants providing services.^34,35,38,39^  • Managing workforce fatigue through task load interventions and biomathematical models.^40,41^  • Rural primary care recruitment activities including schemes for returners, retainer, and re-entry schemes, international recruitment, specialised recruiters, and other support professional and wellbeing support methods.^42,43^  • Ongoing training programmes for PHC professionals in LMICs.^44^  • Financial interventions and movement restrictions to manage the movement of health workers between public and private organisations in LMICs.^46^ |
| Service Delivery: Infrastructure | • Arrangements involving physical infrastructure that ensure people can access to primary care services.  • Policies to ensure that primary care services remain responsive during and following a health emergency or disaster. | • Changing the size of primary care facilities.^50^ |
| Service Delivery: Information Technology |  | • Exploring the effects of mHealth and telemedicine on secondary care use.^54–56^  • Understanding the factors which impact the success of a telehealth programme.^54^  • Using interactive telemedicine and telephone consultations in general practice.^55^  • Hosting laboratory testing in primary care settings.^51^ |
| Service Delivery: Patient supports | • Care coordination and disease management.^120^  • Size, placement, and transport links for health facilities, and services available from health facilities.^120^  • Limited review evidence on: Organisational-level implementation strategies and strategies that addressed characteristics of the wider context in primary care.^123^ | • Applying diversion strategies in general practice settings, providing walk-in centres and general practice co-operatives as an alternative care pathway to emergency department, and triage systems in pre-hospital emergency medical services.^71–74^  • Using primary care service interventions and interdisciplinary team-based models of care to limit hospital admissions.^4,65^  • Putting triage liaison physicians and primary care professionals in emergency department settings.^78,79^  • Strategies to address frequent use of primary care.^84^  • Integrated care models in high-, middle-, and low-income countries.^91,92^  • Primary care referrals to non-clinical support measures (e.g. social prescribing).^94^  • Medication synchronisation and medication review.^101,103^  • Reminder and feedback interventions for medication adherence and quality improvement strategies to reduce antibiotic prescribing.^104,105^ (RL)  • Clinical prediction rules and tools to support decision-making and how they address inappropriate admissions and prescribing.^59^  • Changes to primary care consultation length and organisational culture.^48,87^  • Managing situations where health workers hold two or more jobs (dual practice).^49^ |
| Governance | • Governance arrangements in low income countries.^124^  • Authority and accountability for health policies, healthcare organisations, multi-institutional arrangements, commercial medical products, and quality of practice; training and licensing of health professionals. ^124^ | • Managerial supervision of primary care providers in low- and middle-income countries.^109^  • Public sector regulation, training, or co-ordination of the private for-profit health sector in LMICs.^110^  • Patient engagement in decision-making in emergency departments and in interventions for clinical practice adherence.^111,112^ |

***Systematic reviews needed****: topics for which we did not find any systematic review, based on issues highlighted by included reviews and gaps identified based on our taxonomy (see Table 1).* ***Primary research needed****: topics for research noted in included reviews.*

# References

1 Hone T, Lee JT, Majeed A, Conteh L, Millett C. Does charging different user fees for primary and secondary care affect first-contacts with primary healthcare? A systematic review. *Health Policy Plan* 2017; **32**: 723–31.

2 Park Y, Raza S, George A, Agrawal R, Ko J. The effect of formulary restrictions on patient and payer outcomes: A systematic literature review. J. Manag. Care Spec. Pharm. 2017. DOI:10.18553/jmcp.2017.23.8.893.

3 Green CJ, Maclure M, Fortin PM, Ramsay CR, Aaserud M, Bardal S. Pharmaceutical policies: effects of restrictions on reimbursement. *Cochrane Database Syst Rev* 2010. DOI:10.1002/14651858.CD008654.

4 Carter R, Riverin B, Levesque J-F, Gariepy G, Quesnel-Vallée A. The impact of primary care reform on health system performance in Canada: a systematic review. *BMC Health Serv Res* 2016; **16**: 324.

5 Rashidian A, Omidvari AH, Vali Y, Sturm H, Oxman AD. Pharmaceutical policies: effects of financial incentives for prescribers. *Cochrane Database Syst Rev* 2015. DOI:10.1002/14651858.CD006731.pub2.

6 Brocklehurst P, Price J, Glenny AM, *et al.* The effect of different methods of remuneration on the behaviour of primary care dentists. *Cochrane Database Syst Rev* 2013. DOI:10.1002/14651858.CD009853.pub2.

7 Yuan B, He L, Meng Q, Jia L. Payment methods for outpatient care facilities. *Cochrane Database Syst Rev* 2017. DOI:10.1002/14651858.CD011153.pub2.

8 Gillam SJ, Siriwardena AN, Steel N. Pay-for-Performance in the United Kingdom: Impact of the Quality and Outcomes Framework—A Systematic Review. *Ann Fam Med*  2012; **10**: 461–8.

9 Houle S, McAlister F, Jackevicius C, Chuck A, Tsuyuki R. Does performance-based remuneration for individual health care practitioners affect patient care? A systematic review. *Ann Intern Med* 2012; **157**: 889–99.

10 Van Herck P, De Smedt D, Annemans L, Remmen R, Rosenthal MB, Sermeus W. Systematic review: Effects, design choices, and context of pay-for-performance in health care. *BMC Health Serv Res* 2010; **10**: 247.

11 Gosden T, Forland F, Kristiansen I, *et al.* Capitation, salary, fee‐for‐service and mixed systems of payment: effects on the behaviour of primary care physicians. *Cochrane Database Syst Rev* 2000. DOI:10.1002/14651858.CD002215.

12 Petersen LA, Woodard LD, Urech T, Daw C, Sookanan S. Does Pay-for-Performance Improve the Quality of Health Care?Effectiveness of Pay-for-Performance. *Ann Intern Med* 2006; **145**: 265–72.

13 Odendaal WA, Ward K, Uneke J, *et al.* Contracting out to improve the use of clinical health services and health outcomes in low‐ and middle‐income countries. *Cochrane Database Syst Rev* 2018. DOI:10.1002/14651858.CD008133.pub2.

14 Nijmeijer KJ, Fabbricotti IN, Huijsman R. Is franchising in health care valuable? A systematic review. *Health Policy Plan* 2013; **29**: 164–76.

15 Koehlmoos TP, Gazi R, Hossain SS, Zaman K. The effect of social franchising on access to and quality of health services in low‐ and middle‐income countries. *Cochrane Database Syst Rev* 2009. DOI:10.1002/14651858.CD007136.pub2.

16 Abdulwahid MA, Booth A, Kuczawski M, Mason SM. The impact of senior doctor assessment at triage on emergency department performance measures: systematic review and meta-analysis of comparative studies. *Emerg Med J* 2016; **33**: 504 LP – 513.

17 Cabilan CJ, Boyde M. A systematic review of the impact of nurse-initiated medications in the emergency department. *Australas Emerg Nurs J* 2017; **20**: 53–62.

18 Martin-Misener R, Harbman P, Donald F, *et al.* Cost-effectiveness of nurse practitioners in primary and specialised ambulatory care: systematic review. *BMJ Open* 2015; **5**: e007167.

19 Gielen SC, Dekker J, Francke AL, Mistiaen P, Kroezen M. The effects of nurse prescribing: A systematic review. *Int J Nurs Stud* 2014; **51**: 1048–61.

20 Xue Y, Ye Z, Brewer C, Spetz J. Impact of state nurse practitioner scope-of-practice regulation on health care delivery: Systematic review. *Nurs Outlook* 2016; **64**: 71–85.

21 Chan RJ, Marx W, Bradford N, *et al.* Clinical and economic outcomes of nurse-led services in the ambulatory care setting: A systematic review. *Int J Nurs Stud* 2018; **81**: 61–80.

22 Laurant M, van der Biezen M, Wijers N, Watananirun K, Kontopantelis E, van Vught A. Nurses as substitutes for doctors in primary care. *Cochrane Database Syst Rev* 2018. DOI:10.1002/14651858.CD001271.pub3.

23 WHO. Task Shifting. Global Recomendations and Guidelines. *World Heal Organ* 2008. DOI:10.1080/17441692.2011.552067.

24 Martínez-González NA, Djalali S, Tandjung R, *et al.* Substitution of physicians by nurses in primary care: a systematic review and meta-analysis. *BMC Health Serv Res* 2014; **14**: 214.

25 Donald F, Kilpatrick K, Reid K, *et al.* Hospital to community transitional care by nurse practitioners: A systematic review of cost-effectiveness. *Int J Nurs Stud* 2015; **52**: 436–51.

26 Swan M, Ferguson S, Chang A, Larson E, Smaldone A. Quality of primary care by advanced practice nurses: a systematic review. *Int J Qual Heal Care* 2015; **27**: 396–404.

27 Jennings N, Clifford S, Fox AR, O’Connell J, Gardner G. The impact of nurse practitioner services on cost, quality of care, satisfaction and waiting times in the emergency department: A systematic review. *Int J Nurs Stud* 2015; **52**: 421–35.

28 Randall S, Crawford T, Currie J, River J, Betihavas V. Impact of community based nurse-led clinics on patient outcomes, patient satisfaction, patient access and cost effectiveness: A systematic review. *Int J Nurs Stud* 2017; **73**: 24–33.

29 de Barra M, Scott CL, Scott NW, *et al.* Pharmacist services for non‐hospitalised patients. *Cochrane Database Syst Rev* 2018. DOI:10.1002/14651858.CD013102.

30 Paudyal V, Watson MC, Sach T, *et al.* Are pharmacy-based minor ailment schemes a substitute for other service providers? *Br J Gen Pract* 2013; **63**: e472 LP-e481.

31 Tan ECK, Stewart K, Elliott RA, George J. Pharmacist services provided in general practice clinics: A systematic review and meta-analysis. *Res Soc Adm Pharm* 2014; **10**: 608–22.

32 Okumura LM, Rotta I, Correr CJ. Assessment of pharmacist-led patient counseling in randomized controlled trials: a systematic review. *Int J Clin Pharm* 2014; **36**: 882–91.

33 Pande S, Hiller JE, Nkansah N, Bero L. The effect of pharmacist‐provided non‐dispensing services on patient outcomes, health service utilisation and costs in low‐ and middle‐income countries. *Cochrane Database Syst Rev* 2013. DOI:10.1002/14651858.CD010398.

34 Doan Q, Sabhaney V, Kissoon N, Sheps S, Singer J. A systematic review: The role and impact of the physician assistant in the emergency department. *Emerg Med Australas* 2011; **23**: 7–15.

35 Halter M, Drennan V, Chattopadhyay K, *et al.* The contribution of Physician Assistants in primary care: a systematic review. *BMC Health Serv Res* 2013; **13**: 223.

36 Pennington M, Visram S, Donaldson C, *et al.* Cost-effectiveness of health-related lifestyle advice delivered by peer or lay advisors: synthesis of evidence from a systematic review. *Cost Eff Resour Alloc* 2013; **11**: 30.

37 Vaughan K, Kok MC, Witter S, Dieleman M. Costs and cost-effectiveness of community health workers: evidence from a literature review. *Hum Resour Health* 2015; **13**: 71.

38 Saxon RL, Gray MA, Oprescu FI. Extended roles for allied health professionals: an updated systematic review of the evidence. *J Multidiscip Healthc* 2014; **7**: 479–88.

39 Dyer TA, Brocklehurst P, Glenny AM, *et al.* Dental auxiliaries for dental care traditionally provided by dentists. *Cochrane Database Syst Rev* 2014. DOI:10.1002/14651858.CD010076.pub2.

40 Studnek JR, Infinger AE, Renn ML, *et al.* Effect of Task Load Interventions on Fatigue in Emergency Medical Services Personnel and Other Shift Workers: A Systematic Review. *Prehospital Emerg Care* 2018; **22**: 81–8.

41 James FO, Waggoner LB, Weiss PM, *et al.* Does Implementation of Biomathematical Models Mitigate Fatigue and Fatigue-related Risks in Emergency Medical Services Operations? A Systematic Review. *Prehospital Emerg Care* 2018; **22**: 69–80.

42 Johnson GE, Wright FC, Foster K. The impact of rural outreach programs on medical students’ future rural intentions and working locations: a systematic review. *BMC Med Educ* 2018; **18**: 196.

43 Verma P, Ford JA, Stuart A, Howe A, Everington S, Steel N. A systematic review of strategies to recruit and retain primary care doctors. *BMC Health Serv Res* 2016; **16**: 126.

44 O’Brien MA, Rogers S, Jamtvedt G, *et al.* Educational outreach visits: effects on professional practice and health care outcomes. *Cochrane Database Syst Rev* 2007. DOI:10.1002/14651858.CD000409.pub2.

45 Hedden L, Barer ML, Cardiff K, McGrail KM, Law MR, Bourgeault IL. The implications of the feminization of the primary care physician workforce on service supply: a systematic review. *Hum Resour Health* 2014; **12**: 32.

46 Rutebemberwa E, Kinengyere AA, Ssengooba F, Pariyo GW, Kiwanuka SN. Financial interventions and movement restrictions for managing the movement of health workers between public and private organizations in low‐ and middle‐income countries. *Cochrane Database Syst Rev* 2014. DOI:10.1002/14651858.CD009845.pub2.

47 Baker R, Camosso‐Stefinovic J, Gillies C, *et al.* Tailored interventions to address determinants of practice. *Cochrane Database Syst Rev* 2015. DOI:10.1002/14651858.CD005470.pub3.

48 Parmelli E, Flodgren G, Beyer F, Baillie N, Schaafsma ME, Eccles MP. The effectiveness of strategies to change organisational culture to improve healthcare performance: a systematic review. *Implement Sci* 2011; **6**: 33.

49 Kiwanuka SN, Rutebemberwa E, Nalwadda C, *et al.* Interventions to manage dual practice among health workers. *Cochrane Database Syst Rev* 2011. DOI:10.1002/14651858.CD008405.pub2.

50 Pettigrew LM, Kumpunen S, Mays N, Rosen R, Posaner R. The impact of new forms of large-scale general practice provider collaborations on England’s NHS: a systematic review. *Br J Gen Pract* 2018; **68**: e168 LP-e177.

51 Maillet É, Paré G, Currie LM, *et al.* Laboratory testing in primary care: A systematic review of health IT impacts. *Int J Med Inform* 2018; **116**: 52–69.

52 Iribarren SJ, Cato K, Falzon L, Stone PW. What is the economic evidence for mHealth? A systematic review of economic evaluations of mHealth solutions. *PLoS One* 2017; **12**: e0170581.

53 Akiyama M, Yoo B-K. A Systematic Review of the Economic Evaluation of Telemedicine in Japan. *J Prev Med Public Heal* 2016; **49**: 183–96.

54 Kalankesh LR, Pourasghar F, Nicholson L, Ahmadi S, Hosseini M. Effect of Telehealth Interventions on Hospitalization Indicators: A Systematic Review. *Perspect Heal Inf Manag* 2016; **13**: 1h-1h.

55 Flodgren G, Rachas A, Farmer AJ, Inzitari M, Shepperd S. Interactive telemedicine: effects on professional practice and health care outcomes. *Cochrane Database Syst Rev* 2015. DOI:10.1002/14651858.CD002098.pub2.

56 Downes MJ, Mervin MC, Byrnes JM, Scuffham PA. Telephone consultations for general practice: a systematic review. *Syst Rev* 2017; **6**: 128.

57 Estai M, Kanagasingam Y, Tennant M, Bunt S. A systematic review of the research evidence for the benefits of teledentistry. *J Telemed Telecare* 2017; **24**: 147–56.

58 Wade VA, Karnon J, Elshaug AG, Hiller JE. A systematic review of economic analyses of telehealth services using real time video communication. *BMC Health Serv Res* 2010; **10**: 233.

59 Wallace E, Uijen MJM, Clyne B, *et al.* Impact analysis studies of clinical prediction rules relevant to primary care: a systematic review. *BMJ Open* 2016; **6**: e009957.

60 Souza NM, Sebaldt RJ, Mackay JA, *et al.* Computerized clinical decision support systems for primary preventive care: A decision-maker-researcher partnership systematic review of effects on process of care and patient outcomes. *Implement Sci* 2011; **6**: 87.

61 Randell R, Mitchell N, Dowding D, Cullum N, Thompson C. Effects of computerized decision support systems on nursing performance and patient outcomes: a systematic review. *J Health Serv Res Policy* 2007; **12**: 242–51.

62 Rudin RS, Motala A, Goldzweig CL, Shekelle PG. Usage and effect of health information exchange: a systematic review. *Ann Intern Med* 2014; **161**: 803–11.

63 Mold F, de Lusignan S, Sheikh A, *et al.* Patients’ online access to their electronic health records and linked online services: a systematic review in primary care. *Br J Gen Pract* 2015; **65**: e141 LP-e151.

64 Mitchell E, Sullivan F. A descriptive feast but an evaluative famine: systematic review of published articles on primary care computing during 1980-97. *BMJ* 2001; **322**: 279–82.

65 Crawford J, Cooper S, Cant R, DeSouza R. The impact of walk-in centres and GP co-operatives on emergency department presentations: A systematic review of the literature. *Int Emerg Nurs* 2017; **34**: 36–42.

66 Raven MC, Kushel M, Ko MJ, Penko J, Bindman AB. The Effectiveness of Emergency Department Visit Reduction Programs: A Systematic Review. *Ann Emerg Med* 2016; **68**: 467-483.e15.

67 Morgan SR, Chang AM, Alqatari M, Pines JM. Non–emergency department interventions to reduce ED utilization: a systematic review. *Acad Emerg Med* 2013; **20**: 969–85.

68 Flores-Mateo G, Violan-Fors C, Carrillo-Santisteve P, Peiró S, Argimon J-M. Effectiveness of Organizational Interventions to Reduce Emergency Department Utilization: A Systematic Review. *PLoS One* 2012; **7**: e35903.

69 Huntley A, Lasserson D, Wye L, *et al.* Which features of primary care affect unscheduled secondary care use? A systematic review. *BMJ Open* 2014; **4**: e004746.

70 Brainard JS, Ford JA, Steel N, Jones AP. A systematic review of health service interventions to reduce use of unplanned health care in rural areas. *J Eval Clin Pract* 2016; **22**: 145–55.

71 Lidal IB, Holte HH, Vist GE. Triage systems for pre-hospital emergency medical services - a systematic review. *Scand J Trauma Resusc Emerg Med* 2013; **21**: 28.

72 Kirkland SW, Soleimani A, Rowe BH, Newton AS. A systematic review examining the impact of redirecting low-acuity patients seeking emergency department care: is the juice worth the squeeze? *Emerg Med J* 2019; **36**: 97 LP – 106.

73 Ismail SA, Gibbons DC, Gnani S. Reducing inappropriate accident and emergency department attendances: *Br J Gen Pract* 2013; **63**: e813 LP-e820.

74 Morley C, Unwin M, Peterson GM, Stankovich J, Kinsman L. Emergency department crowding: A systematic review of causes, consequences and solutions. *PLoS One* 2018; **13**: e0203316.

75 Rowe BH, Villa-Roel C, Guo X, *et al.* The Role of Triage Nurse Ordering on Mitigating Overcrowding in Emergency Departments: A Systematic Review. *Acad Emerg Med* 2011; **18**: 1349–57.

76 Galipeau J, Pussegoda K, Stevens A, *et al.* Effectiveness and Safety of Short-stay Units in the Emergency Department: A Systematic Review. *Acad Emerg Med* 2015; **22**: 893–907.

77 Bullard MJ, Villa-Roel C, Guo X, *et al.* The role of a rapid assessment zone/pod on reducing overcrowding in emergency departments: a systematic review. *Emerg Med J* 2012; **29**: 372 LP – 378.

78 Gonçalves‐Bradley D, Khangura JK, Flodgren G, Perera R, Rowe BH, Shepperd S. Primary care professionals providing non‐urgent care in hospital emergency departments. *Cochrane Database Syst Rev* 2018. DOI:10.1002/14651858.CD002097.pub4.

79 Rowe BH, Guo X, Villa-Roel C, *et al.* The Role of Triage Liaison Physicians on Mitigating Overcrowding in Emergency Departments: A Systematic Review. *Acad Emerg Med* 2011; **18**: 111–20.

80 Soril LJJ, Leggett LE, Lorenzetti DL, Noseworthy TW, Clement FM. Reducing frequent visits to the emergency department: a systematic review of interventions. *PLoS One* 2015; **10**: e0123660–e0123660.

81 Moe J, Kirkland SW, Rawe E, *et al.* Effectiveness of Interventions to Decrease Emergency Department Visits by Adult Frequent Users: A Systematic Review. *Acad Emerg Med* 2017; **24**: 40–52.

82 Althaus F, Paroz S, Hugli O, *et al.* Effectiveness of Interventions Targeting Frequent Users of Emergency Departments: A Systematic Review. *Ann Emerg Med* 2011; **58**: 41-52.e42.

83 Kumar GS, Klein R. Effectiveness of Case Management Strategies in Reducing Emergency Department Visits in Frequent User Patient Populations: A Systematic Review. *J Emerg Med* 2013; **44**: 717–29.

84 Haroun D, Smits F, van Etten-Jamaludin F, Schene A, van Weert H, ter Riet G. The effects of interventions on quality of life, morbidity and consultation frequency in frequent attenders in primary care: A systematic review. *Eur J Gen Pract* 2016; **22**: 71–82.

85 Rose KD, Ross JS, Horwitz LI. Advanced Access Scheduling Outcomes: A Systematic Review. *JAMA Intern Med* 2011; **171**: 1150–9.

86 Krogsbøll LT, Jørgensen KJ, Gøtzsche PC. General health checks in adults for reducing morbidity and mortality from disease. *Cochrane Database Syst Rev* 2019. DOI:10.1002/14651858.CD009009.pub3.

87 Wilson AD, Childs S, Gonçalves‐Bradley DC, Irving GJ. Interventions to increase or decrease the length of primary care physicians’ consultation. *Cochrane Database Syst Rev* 2016. DOI:10.1002/14651858.CD003540.pub3.

88 Irving G, Neves AL, Dambha-Miller H, *et al.* International variations in primary care physician consultation time: a systematic review of 67 countries. *BMJ Open* 2017; **7**: e017902.

89 Bunn F, Byrne G, Kendall S. Telephone consultation and triage: effects on health care use and patient satisfaction. *Cochrane Database Syst Rev* 2004. DOI:10.1002/14651858.CD004180.pub2.

90 Kaufman BG, Spivack BS, Stearns SC, Song PH, O’Brien EC. Impact of Accountable Care Organizations on Utilization, Care, and Outcomes: A Systematic Review. *Med Care Res Rev* 2017; **76**: 255–90.

91 Dudley L, Garner P. Strategies for integrating primary health services in low‐ and middle‐income countries at the point of delivery. *Cochrane Database Syst Rev* 2011. DOI:10.1002/14651858.CD003318.pub3.

92 Baxter S, Johnson M, Chambers D, Sutton A, Goyder E, Booth A. The effects of integrated care: a systematic review of UK and international evidence. *BMC Health Serv Res* 2018; **18**: 350.

93 Elrashidi MY, Mohammed K, Bora PR, *et al.* Co-located specialty care within primary care practice settings: A systematic review and meta-analysis. *Healthcare* 2018; **6**: 52–66.

94 Bickerdike L, Booth A, Wilson PM, Farley K, Wright K. Social prescribing: less rhetoric and more reality. A systematic review of the evidence. *BMJ Open* 2017; **7**. http://bmjopen.bmj.com/content/7/4/e013384.abstract.

95 Gruen RL, Weeramanthri TS, Knight SS, Bailie RS. Specialist outreach clinics in primary care and rural hospital settings. *Cochrane Database Syst Rev* 2003. DOI:10.1002/14651858.CD003798.pub2.

96 Akbari A, Mayhew A, Al‐Alawi MA, *et al.* Interventions to improve outpatient referrals from primary care to secondary care. *Cochrane Database Syst Rev* 2008. DOI:10.1002/14651858.CD005471.pub2.

97 Faulkner A, Mills N, Bainton D, *et al.* A systematic review of the effect of primary care-based service innovations on quality and patterns of referral to specialist secondary care. *Br J Gen Pract* 2003; **53**: 878–84.

98 McNab D, Bowie P, Ross A, MacWalter G, Ryan M, Morrison J. Systematic review and meta-analysis of the effectiveness of pharmacist-led medication reconciliation in the community after hospital discharge. *BMJ Qual &amp;amp; Saf* 2018; **27**: 308 LP – 320.

99 Hatah E, Braund R, Tordoff J, Duffull SB. A systematic review and meta-analysis of pharmacist-led fee-for-services medication review. *Br J Clin Pharmacol* 2014; **77**: 102–15.

100 de Bont EGPM, Alink M, Falkenberg FCJ, Dinant G-J, Cals JWL. Patient information leaflets to reduce antibiotic use and reconsultation rates in general practice: a systematic review. *BMJ Open* 2015; **5**: e007612.

101 Nguyen E, Sobieraj DM. The impact of appointment-based medication synchronization on medication taking behaviour and health outcomes: A systematic review. *J Clin Pharm Ther* 2017; **42**: 404–13.

102 West LM, Diack L, Cordina M, Stewart D. A systematic review of the literature on ‘medication wastage’: an exploration of causative factors and effect of interventions. *Int J Clin Pharm* 2014; **36**: 873–81.

103 Royal S, Smeaton L, Avery AJ, Hurwitz B, Sheikh A. Interventions in primary care to reduce medication related adverse events and hospital admissions: systematic review and meta-analysis. *Qual Saf Heal Care* 2006; **15**: 23 LP – 31.

104 Bennett JW, Glasziou PP. Computerised reminders and feedback in medication management: a systematic review of randomised controlled trials. *Med J Aust* 2003; **178**: 217–22.

105 Ranji SR, Steinman MA, Shojania KG, Gonzales R. Interventions to Reduce Unnecessary Antibiotic Prescribing: A Systematic Review and Quantitative Analysis. *Med Care* 2008; **46**. https://journals.lww.com/lww-medicalcare/Fulltext/2008/08000/Interventions_to_Reduce_Unnecessary_Antibiotic.12.aspx.

106 Reddy S, Jones P, Shanthanna H, Damarell R, Wakerman J. A Systematic Review of the Impact of Healthcare Reforms on Access to Emergency Department and Elective Surgery Services: 1994–2014. *Int J Heal Serv* 2017; **48**: 81–105.

107 Watkins K, Wood H, Schneider CR, Clifford R. Effectiveness of implementation strategies for clinical guidelines to community pharmacy: a systematic review. *Implement Sci* 2015; **10**: 151.

108 Losier M, Ramsey TD, Wilby KJ, Black EK. A Systematic Review of Antimicrobial Stewardship Interventions in the Emergency Department. *Ann Pharmacother* 2017; **51**: 774–90.

109 Bosch‐Capblanch X, Liaqat S, Garner P. Managerial supervision to improve primary health care in low‐ and middle‐income countries. *Cochrane Database Syst Rev* 2011. DOI:10.1002/14651858.CD006413.pub2.

110 Wiysonge CS, Abdullahi LH, Ndze VN, Hussey GD. Public stewardship of private for‐profit healthcare providers in low‐ and middle‐income countries. *Cochrane Database Syst Rev* 2016. DOI:10.1002/14651858.CD009855.pub2.

111 Flynn D, Knoedler MA, Hess EP, *et al.* Engaging Patients in Health Care Decisions in the Emergency Department Through Shared Decision-making: A Systematic Review. *Acad Emerg Med* 2012; **19**: 959–67.

112 Fønhus MS, Dalsbø TK, Johansen M, Fretheim A, Skirbekk H, Flottorp SA. Patient‐mediated interventions to improve professional practice. *Cochrane Database Syst Rev* 2018. DOI:10.1002/14651858.CD012472.pub2.

113 Park Y, Raza S, George A, Agrawal R, Ko J. The Effect of Formulary Restrictions on Patient and Payer Outcomes: A Systematic Literature Review. *J Manag Care Spec Pharm* 2017; **23**: 893–901.

114 Brocklehurst P, Price J, Glenny AM, *et al.* The effect of different methods of remuneration on the behaviour of primary care dentists. *Cochrane Database Syst Rev* 2013. DOI:10.1002/14651858.CD009853.pub2.

115 Flodgren G, Gonçalves‐Bradley DC, Pomey MP. External inspection of compliance with standards for improved healthcare outcomes. *Cochrane Database Syst Rev* 2016. DOI:10.1002/14651858.CD008992.pub3.

116 Ward MM, Jaana M, Natafgi N. Systematic review of telemedicine applications in emergency rooms. *Int J Med Inform* 2015; **84**: 601–16.

117 Wiysonge CS, Paulsen E, Lewin S, *et al.* Financial arrangements for health systems in low-income countries: An overview of systematic reviews. Cochrane Database Syst. Rev. 2017; **2017**. DOI:10.1002/14651858.CD011084.pub2.

118 Eijkenaar F, Emmert M, Scheppach M, Schöffski O. Effects of pay for performance in health care: A systematic review of systematic reviews. *Health Policy (New York)* 2013; **110**: 115–30.

119 Flodgren G, Eccles MP, Shepperd S, Scott A, Parmelli E, Beyer FR. An overview of reviews evaluating the effectiveness of financial incentives in changing healthcare professional behaviours and patient outcomes. *Cochrane Database Syst Rev* 2011. DOI:10.1002/14651858.CD009255.

120 Ciapponi A, Lewin S, Herrera CA, *et al.* Delivery arrangements for health systems in low-income countries: An overview of systematic reviews. Cochrane Database Syst. Rev. 2017; **2017**. DOI:10.1002/14651858.CD011083.pub2.

121 Mossialos E, Naci H, Courtin E. Expanding the role of community pharmacists: Policymaking in the absence of policy-relevant evidence? *Health Policy (New York)* 2013; **111**: 135–48.

122 Scott K, Beckham SW, Gross M, *et al.* What do we know about community-based health worker programs? A systematic review of existing reviews on community health workers. *Hum Resour Health* 2018; **16**: 39.

123 Lau R, Stevenson F, Ong BN, *et al.* Achieving change in primary care—effectiveness of strategies for improving implementation of complex interventions: systematic review of reviews. *BMJ Open* 2015; **5**: e009993.

124 Herrera CA, Lewin S, Paulsen E, *et al.* Governance arrangements for health systems in low-income countries: An overview of systematic reviews. Cochrane Database Syst. Rev. 2017; **2017**. DOI:10.1002/14651858.CD011085.pub2.

1. There may be some instances in which ‘findings’ and ‘focus’ of included reviews do not perfectly align. This is because some reviews have a focus which covers multiple categories in our taxonomy. For example, a review may cover both “Financing” and “Service Delivery: Support measures”. [↑](#footnote-ref-2)
2. *Positive* impacts refer to “desirable effects, such as reduced costs or unnecessary healthcare utilisation, identified with no clear evidence of any undesirable effects, such as worse health outcomes”. [↑](#footnote-ref-3)
